# In-Depth Mass Spectrometry Analysis Reveals the Plasma Proteomic and N-Glycoproteomic Impact of an Amish-Enriched Cardioprotective Variant in *B4GALT1*

**DOI:** 10.1016/j.mcpro.2023.100595

**Published:** 2023-06-15

**Authors:** Yunlong Zhao, Shruti Nayak, Shivkumar Raidas, Lili Guo, Giusy Della Gatta, Sujeethraj Koppolu, Gabor Halasz, May E. Montasser, Alan R. Shuldiner, Yuan Mao, Ning Li

**Affiliations:** 1 Analytical Chemistry Group, Regeneron Pharmaceuticals, Inc, Tarrytown, New York, USA; 2 Regeneron Genetics Center, LLC, Tarrytown, New York, USA; 3 Molecular Profiling and Data Science, Regeneron Pharmaceuticals, Inc, Tarrytown, New York, USA; 4 Division of Endocrinology, Diabetes and Nutrition and Program for Personalized and Genomic Medicine, Department of Medicine, University of Maryland School of Medicine, Baltimore, Maryland, USA

**Keywords:** Amish-enriched cardioprotective variant, coagulation pathway, human plasma, lipid metabolism, low-density lipoprotein, quantitative N-glycoproteomics, site-specific glycan traits analysis, tandem mass tag, β-1,4-galactosyltransferase 1

## Abstract

*B4GALT1* encodes β-1,4-galactosyltransferase 1, an enzyme that plays a major role in glycan synthesis in the Golgi apparatus by catalyzing the addition of terminal galactose. Studies increasingly suggest that B4GALT1 may be involved in the regulation of lipid metabolism pathways. Recently, we discovered a single-site missense variant Asn352Ser (N352S) in the functional domain of B4GALT1 in an Amish population, which decreases the level of LDL-cholesterol (LDL-c) as well as the protein levels of ApoB, fibrinogen, and IgG in the blood. To systematically evaluate the effects of this missense variant on protein glycosylation, expression, and secretion, we developed a nano-LC-MS/MS-based platform combined with TMT-labeling for in-depth quantitative proteomic and glycoproteomic analyses in the plasma of individuals homozygous for the *B4GALT1* missense variant N352S *versus* non-carriers (n = 5 per genotype). A total of 488 secreted proteins in the plasma were identified and quantified, 34 of which showed significant fold changes in protein levels between N352S homozygotes and non-carriers. We determined N-glycosylation profiles from 370 glycosylation sites in 151 glycoproteins and identified ten proteins most significantly associated with decreased galactosylation and sialyation in *B4GALT1* N352S homozygotes. These results further support that *B4GALT1* N352S alters the glycosylation profiles of a variety of critical target proteins, thus governing the functions of these proteins in multiple pathways, such as those involved in lipid metabolism, coagulation, and the immune response.

## Highlights

• Differential proteome and N-glycoproteome between B4GALT1 N352S vs. WT human plasma.
• An increase in incomplete galacosylation with minor change in global protein levels.
• Glycan trait interpretation provided insights into the impact on terminal saccharides.
• Shed light on the relationship of key proteins and their N-glycosylation in pathways.

## Graphical Abstract

**Figure:**
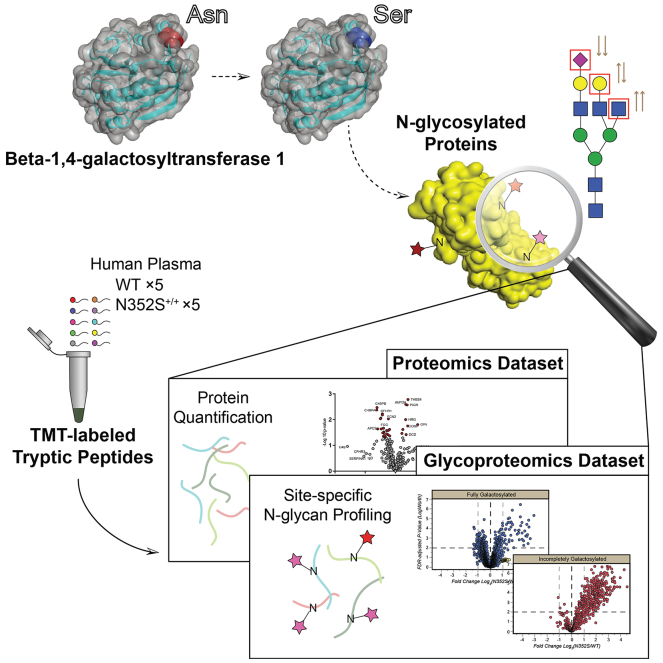

## In Brief

The proteome and N-glycoproteome of WT and N352S B4GALT1 plasma samples from Amish individuals were studied. While only a few proteins exhibited altered expression levels, the reduced activity of *B4GALT1* in N352S samples resulted in more incompletely galactosylated glycoforms across a wide range of N-glycosylation sites. Furthermore, several glycan traits involving terminal saccharides were determined, revealing that the N352S mutation in B4GALT1 may impact relevant pathways by altering the N-glycosylation profiles of key proteins, including apolipoprotein B and fibrinogen.

Glycosylation profiles are controlled by a series of glycan synthesis enzymes. Changes in the abundance and activity of these enzymes may alter the glycosylation profiles of a population of substrate glycoproteins. β-1,4-galactosyltransferase I (B4GALT1) is a glycan synthesis enzyme that catalyzes the addition of β-galactose to core N-glycan structures during stepwise protein glycosylation in the Golgi apparatus. Recently, we have reported the enrichment of a naturally occurring missense variant of *B4GALT1* encoding a single-site mutation, N352S, in Amish subjects (1). N352 is located in the functional domain of *B4GALT1* and therefore might allosterically decrease the enzyme’s activity. Indeed, we have shown that the profiles of the glycans released from whole plasma proteins in subjects homozygous for this variant have lower galactosylation levels compared with non-carriers (*B4GALT1* WT). In addition, the *B4GALT1* N352S genotype is associated with diminished levels of LDL-cholesterol (LDL-c) and fibrinogen as also phenocopied in an animal model (1). These findings underscore the relationship between lipid metabolism and glycosylation. As shown in other studies, there are many glycoproteins that affect circulating lipid pathways, including cholesterol synthesis, lipoprotein assembly, transport, and recycling. Their functions can be affected by the level of N- or O-linked glycosylation and the relative distribution of diverse glycan structures (2). Intriguingly, N352S only partially decreases the activity of B4GALT1, and data from both human and animal models did not identify any severe phenotype that might be associated with this variant (1). In contrast, complete depletion or other loss-of-function mutations of *B4GALT1* lead to a severe congenital glycosylation type II disorder (3). Because increased levels of LDL-c and fibrinogen are critical markers indicating high cardiovascular disease risk (4, 5), tuning LDL-c and fibrinogen levels through modulating B4GALT1 activity may provide a novel therapeutic strategy.

Because N-glycan elongation by B4GALT1 occurs mainly in the Golgi apparatus, which plays a central role in protein secretion, the altered activity of B4GALT1 may result in changes in glycosylation profiles and protein abundance for a variety of glycoproteins in the blood (6). We previously reported a lower level of galactosylated N-glycans for several highly abundant plasma glycoproteins purified individually from *B4GALT1* N352S homozygotes (1), including apolipoprotein B-100 (ApoB), fibrinogen, immunoglobulin G (IgG), and transferrin; however, a proteomic analysis had not been performed to identify other plasma glycoproteins whose glycoprofiles and secretion levels might be affected. Beyond glycosylation-associated secretion, changes in glycosylation profiles may also affect structure-associated protein functions. Functional changes in certain glycoproteins may further affect the abundance of other proteins within entire pathways, regardless of their glycosylation status. Therefore, we conducted an in-depth (glyco)proteomic analysis of plasma samples to identify the main affected glycoproteins and glycosylation sites in the glycosylation profile of N352 homozygotes, as well as to systematically evaluate differences in protein levels to highlight possible effects on protein secretion and function. Together, the findings enabled the study of the mechanisms through which related pathways are affected in response to N352S-induced B4GALT1 functional suppression. In addition, protein glycosylation is known to affect many other activities in the blood, such as the coagulation cascade and immune response. Therefore, a proteome scale analysis would aid in the evaluation of any off-target effects during the development of therapeutics.

Isobaric tag labeling (*e.g.*, iTRAQ and TMT), a commonly used strategy for quantitative proteomics and glycoproteomics, enables multiplexing in sample preparation as well as LC-MS/MS analysis. Compared with the label-free strategy, the use of isobaric tags effectively decreases the required sample amount, enhances analytical throughput, and increases quantification accuracy. For glycoproteomics with isobaric tag labeling, the mass spectrometry parameters, including different types of fragmentation techniques, must usually be optimized to generate abundant signals from isotope reporter ions for quantification as well as glycan and backbone fragmentation ions for identification (7, 8). Novel data processing algorithms have been explored to address the challenge of increased complexity in the fragment ions in single MS/MS spectra generated from isobaric tag-labeled glycopeptide precursors (9). The isobaric labeling strategy has been widely used to evaluate glycoproteome remodeling in various sample types through the identification and quantification of intact glycopeptides (10, 11, 12, 13). Quantitative glycoproteomics has emerged as a powerful tool to link the altered function of critical enzymes involved in glycan biosynthesis (such as glycosyltransferases, carbohydrate transporters, and glycosidases) to changes in the profile of glycoproteins and signaling pathways. Several studies have demonstrated the utility of this approach in investigating fucosylation, such as the work of Stadlmann *et al.* (14), who used the TMT strategy to evaluate the effects of *SLC35C1*, encoding a GDP-fucose transporter, on the relative level change of fucosylated peptides in embryonic cells in response to ricin toxicity. Fang, *et al.* (15) employed a synchronous precursor selection-MS3 TMT strategy to investigate changes in cellular fucosylation levels after treatment with a fucosyltransferase inhibitor.

Unlike fucose, which is not involved in glycan chain elongation, galactose and N-acetylgalactosamine (GalNAc), can be commonly found at both N-glycan termini and semi-termini being further capped by saccharides such as sialic acid. Therefore, changes in the synthesis process for these saccharides could affect the distribution of a wide range of N-glycans and O-glycans (16). In the case of B4GALT1, we hypothesized that changes in β-galactosylation status may affect the relative levels of a variety of terminal saccharides, including terminal α-galactose, terminal β-galactose, terminal antennary GlcNAc, terminal GalNAc, and sialic acid. Different terminal saccharides have diverse functions and are often associated with protein recognition and signaling regulation. To our knowledge, sialic acids contribute to acidic charge variants and can affect protein-protein interactions (17); the terminal galactose is associated with the Fc-glycan complement-dependent cytotoxicity activity of immunoglobulin (18), and the cell surface binding of adeno-associated virus 9 (19). The terminal antennary and bisecting GlcNAc is also associated with the activation of the lectin-involved complement pathway (20). Therefore, these terminal saccharides may be considered important benchmarks in evaluating the effects of altered B4GALT1 activity on downstream glycoprotein function. In this work, we applied TMT-labeling proteomic and glycoproteomic approaches to the analysis of human plasma samples from ten Amish individuals with wild-type (WT) *B4GALT1* or homozygous variant (N352S) genotypes. A glycan trait-centric data mining approach was also used to identify differences in N-glycosylation profiles as well as terminal saccharides. We found that complement and coagulation proteins showed modest differences in abundance between genotypes. In addition, profiles of N-glycosylation and terminal saccharides from a variety of glycoproteins in multiple pathways were affected in the *B4GALT1* N352S genotype samples.

## Experimental Procedures

### Experimental Design and Statistical Rationale

**Fig. 1:**
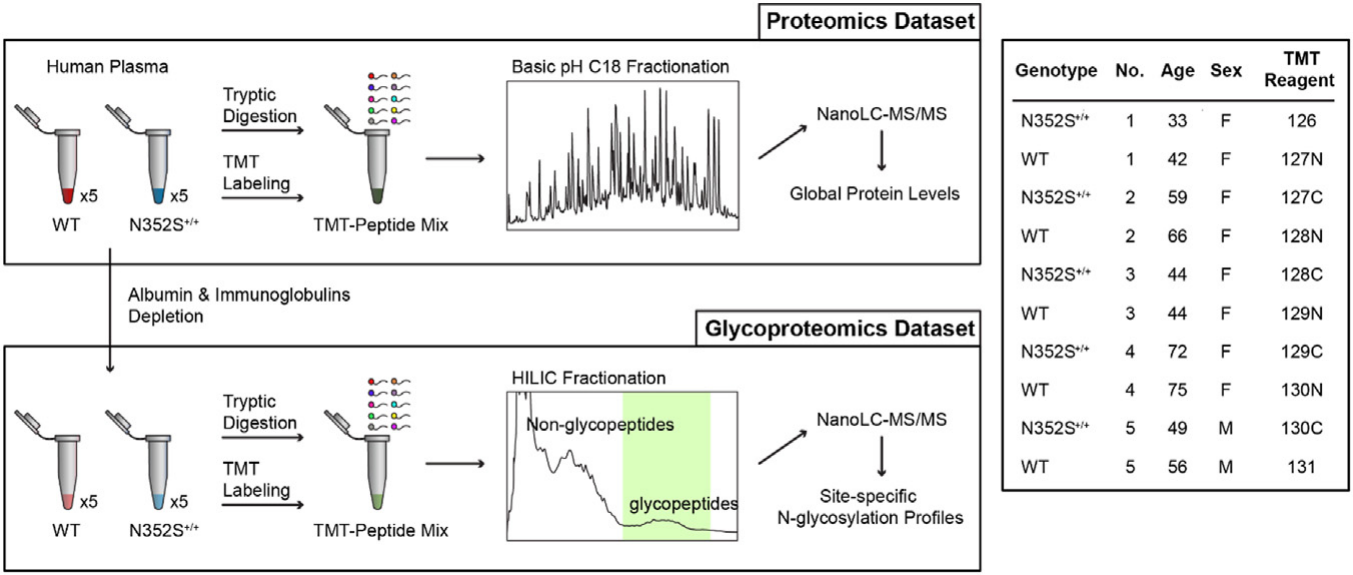
**Workflow of analytical strategy for global proteomics and glycoproteomics**.

This study was approved by the institutional review board at the University of Maryland Baltimore (IRB number HP-00074889). Informed consent was obtained from each study participant. The overall analytical workflow is depicted in Figure 1. Human blood plasma samples were collected from ten Amish individuals, comprising five homozygous for the N352S variant of the *B4GALT1* gene and five with the WT gene, who were matched for age and sex (see Study Population for detailed information). Sample collection was performed by following a standard operation procedure for phlebotomy and blood specimen handling issued by the University of Maryland Baltimore. Five biological replicates from the two genotype groups (WT and N352S) were processed for both proteomics analysis and N-glycoproteomics analysis, detailed in other subsections. Different levels of plasma protein and site-specific glycan between the two genotype groups were evaluated by the fold change in TMT reporter ion intensities followed by ANOVA. To better illustrate the impact of galactosylation-related events and reduce the signal variations among biological replications, selected glycan traits from each glycosylation site were also calculated based on the normalized level of individual glycopeptide from the same glycosylation site, detailed in the subsection Glycan Trait Calculation.

### Study Population

The Old Order Amish population of Lancaster County, PA, immigrated to North America from Central Europe in the early 1700s. There are currently around 40,000 Old Order Amish individuals in the Lancaster area. Investigators at the University of Maryland School of Medicine have been studying the genetic determinants of cardiometabolic health in this population since 1993. To date, about 10,000 Amish adults have participated in one or more studies as part of the Amish Complex Disease Research Program (ref: Amish Research Program http://www.medschool.umaryland.edu/endocrinology/Amish-Research-Program/). These studies generated a genotype database that was used to identify matched opposite homozygotes for the missense variant (rs551564683, p.Asn352Ser) in *B4GALT1*, who were either siblings or closely related same-sex individuals based on the kinship coefficient. They were also matched for age (within 5 years) and carrier status for APOB p.Arg3527Gln genotype. We excluded pregnant women and anyone taking statins, glucocorticoids (*e.g.*, Prednisone), warfarin, and clopidogrel (and other medications that affect clotting). None of the participants were taking lipid-lowering medication. The basic and clinical characteristics of the study samples are presented in supplemental Table S1 and Figure 1.

### Sample Preparation for Proteomic Analysis

The plasma protein concentration in each sample was determined with BCA assays (Thermo Fisher Scientific). Plasma protein (200 μg) was directly denatured and reduced in 8 M urea and 5 mM dithiothreitol at 37 °C for 1 h and then incubated with 15 mM iodoacetamide in the dark at 25 °C for 30 min. The reduced and alkylated samples were first digested by rLys-C (Promega) at a 1:50 enzyme-to-substrate ratio at 37 °C for 2 h. The urea concentration was then decreased to 1 M with 100 mM triethylammonium bicarbonate buffer. The samples were then digested with trypsin at a 1:50 enzyme-to-substrate ratio at 37 °C for another 17 h. Each digested sample was desalted with a Sap-Pak C18 cartridge (Waters). 800 μg of isobaric TMT10plex reagents (Thermo Fisher Scientific) reconstituted in anhydrous ethanol was added to each sample (at a 1:4 peptide-to-TMT reagent ratio) and incubated for 1 h at room temperature. The TMT reaction was stopped by incubation with 8 μl of 5% hydroxylamine for 15 min at room temperature. The samples were then equally pooled and desalted again.

Basic-pH reversed-phase chromatography fractionation was performed with a Waters Acquity UPLC system equipped with a binary pump for mobile phase A (10 mM ammonium formate, pH 8.0) and mobile phase B (95% acetonitrile and 10 mM ammonium formate, pH 8.0) and an automated fraction collector. The pooled TMT-labeled peptides were injected on a Waters XBridge C18 column (4.6 mm I.D. × 25 cm length) and separated by increasing mobile phase B from 15% to 35% within 1 h at a flow rate of 500 μl/min. Fractions were collected every minute throughout the gradient. The fractions were combined into 20 fractions with a concatenation strategy (21). Each fraction was dried and reconstituted in 50 μl of H_2_O with 0.1% FA for nanoLC-MS/MS analysis.

### Sample Preparation for Glycoproteomic Analysis

For the glycoproteomic analysis, the highly abundant albumin and immunoglobulins were first depleted with affinity-based depletion midi columns (Thermo Fisher Scientific). The plasma protein concentration after depletion was determined with BCA assays. Subsequently, 200 μg of protein from each sample was mixed with the glycoprotein standard (produced and purified in-house) in various amounts (WT: 0.8 μg, 0.2 μg, 0.6 μg, 0.4 μg, 0.2 μg; N352S: 2 μg, 0.4 μg, 1 μg, 1.6 μg, 2 μg). The samples were then digested and TMT-labeled with the same procedure as in the proteomic study. The TMT-labeled samples were equally pooled and desalted before HILIC-based glycopeptide enrichment with a previously reported method (14, 22).

Glycopeptides were enriched and fractionated with a Waters Acquity UPLC system equipped with a binary pump for mobile phase A (0.1% trifluoracetic acid) and mobile phase B (100% acetonitrile and 0.1% trifluoracetic acid) and an automated fraction collector. The pooled TMT-labeled peptides were injected into a Tskgel Amide-80 HR column (4.6 mm I.D. × 25 cm length) (Tosoh Bioscience) and separated by decreasing mobile phase B from 80% to 60% over 40 min at a flow rate of 750 μl/min. Fractions were collected every 30 s throughout the gradient. Each fraction potentially containing glycopeptides, according to the UV traces (Fig. 1), was dried and reconstituted in 50 μl water with 0.1% formic acid for nanoLC-MS/MS analysis. The non-glycopeptide-containing fractions were also analyzed with nanoLC-MS/MS to validate the labeling efficiency and intra-group variations in protein levels.

### NanoLC-MS/MS Analysis

An aliquot of each sample was analyzed with a Thermo Scientific Dionex Ultimate RSLC 3000 nanoflow liquid chromatography system coupled to a Thermo Scientific Q Exactive HF-X hybrid mass spectrometer with a Nanoflex electrospray source. The loading buffer and mobile phase A were 0.1% formic acid, and mobile phase B was 80% acetonitrile and 0.1% formic acid. Samples were loaded on a trapping column (75 μm I.D. × 2 cm length) (Thermo Fisher Scientific) for 5 min at a flow rate of 5 μl/min, then were separated on an Acclaimed PepMap C18 column (75 μm I.D. × 25 cm length) with a 6% to 40% gradient of mobile phase B over 125 min at a flow rate of 0.3 μl/min.

The source parameters were set as follows: spray voltage = 2.2 kV, sheath gas flow rate = 0, capillary temperature = 325 °C, and funnel RF level = 50. For the basic-pH fractions of proteomics samples, MS1 resolution: 60,000, scan range: 375 to 1500 m/z, AGC target: 3e6, and maximum ion injection time: 50 ms. After each MS1 scan, high-resolution HCD MS/MS spectra were acquired for the top ten most abundant precursor ions, with MS/MS resolution: 45,000, AGC target: 1e5, maximum ion injection time: 20 ms, isolation window: 2.0 m/z, normalized collision energy: 35, and dynamic exclusion: 15 s. For the HILIC fractions of glycoproteomic samples, MS1 resolution: 120,000, scan range: 375 to 2000 m/z, AGC target: 3e6, and maximum ion injection time: 60 ms. After each MS1 scan, high-resolution HCD MS/MS spectra were acquired for the top ten most abundant precursor ions. MS/MS resolution: 45,000, AGC target: 5e5, maximum ion injection time: 250 ms, isolation window of 0.9 m/z, normalized collision energy: 35, and dynamic exclusion: 10 s.

### Data Processing and Statistical Analysis

For the proteomic dataset, raw MS files from all fractions were processed and merged into a single result file with Proteome Discoverer 2.2 (Thermo Fisher Scientific) for protein identification and quantification using a processing workflow as shown in supplemental Fig. S1. The human reference proteome (containing 90,807) sequences downloaded from the UniProt server (by Dec 2020) was used as the protein sequence database. Oxidation at methionine and acetylation at protein N-terminal were selected as dynamic modifications. Carbamidomethylation at cysteine and TMT6plex at peptide N-terminal and lysine were selected as fixed modifications. Precursor mass tolerance = 10 ppm and fragment mass tolerance = 0.02 Da. 1% false positive rate was applied based on the reversed sequence decoy. Only the proteins with at least two identified peptides were kept. TMT reporter ions for each spectrum were extracted and consolidated for peptide and protein quantification. Protein IDs and quantification results for individual PTMs, peptides and proteins were exported for further normalization in Excel (see detailed information in the Results session).

For glycoproteomics, the same processing workflow and searching parameters were applied for glycopeptide identification and quantification. For an improved capability of glycopeptide identification, a built-in search engine node of Byonic 2.6 (Protein Metrics) was incorporated into the processing workflow to replace Sequest HT (supplemental Fig. S1). In addition to including all the above-mentioned modifications, a glycan database containing 140 unique N-glycan compositions, created according to our in-house human plasma glycomics results, was imported into the Byonic node as dynamic modifications for searching glycoforms of glycopeptides. Peptide-spectrum matches (PSMs) for intact N-glycopeptides were further validated with a score filter (Byonic score > 300, |LogProb| > 2). Manual MS/MS verification was performed for less common glycoforms. All validated PSMs and TMT reporter ion intensity data were exported for further normalization in Excel (see detailed information in the Results session).

Statistical analyses for global protein and glycopeptide level changes were performed using JMP software based on normalized data. For each gene (protein group) or glycoform, the fold change of signal abundance between the two genotype groups (N352S/WT) was evaluated through *t* test (n = 5).

### Glycan Traits Calculation

Selective glycan traits including the rate of occurrence of β-galactosylation on each GlcNAc branch (*%Gal)*, terminal (non-galactosylated) GlcNAc (*%terminalGlcNAc*), terminal β-galactosylation (*%terminalGal*), sialylation per galactose (*%Sia/Gal*), sialylation per branch (*%Sia/Branch*), and fucosylation at the core GlcNAc (*%coreFuc*) for each identified N-glycosylation site were calculated using in-house generated VBA scripts incorporating with the following equation:

$$\%Gal=\frac{\sum_{k=1}^{N}[\#Gal@glc\left(k\right)\times Intensity@glc\left(k\right)]}{\sum_{k=1}^{N}\left[\#GlcNAc@glc\left(k\right)\times Intensity@glc\left(k\right)\right]}$$

$$\%terminalGlcNAc=1-\frac{\sum_{k=1}^{N}[\#Gal@glc\left(k\right)\times Intensity@glc\left(k\right)]}{\sum_{k=1}^{N}\left[\#GlcNAc@glc\left(k\right)\times Intensity@glc\left(k\right)\right]}$$

$$\%terminalGal=\frac{\sum_{k=1}^{N}\{\left[\#Gal@glc\left(k\right)-\#Sia@glc\left(k\right)\right]\times Intensity@glc\left(k\right)\}}{\sum_{k=1}^{N}\left[\#GlcNAc@glc\left(k\right)\times Intensity@glc\left(k\right)\right]}$$

$$\%Sia/Gal=\frac{\sum_{k=1}^{N}[\#Sia@glc\left(k\right)\times Intensity@glc\left(k\right)]}{\sum_{k=1}^{N}\left[\#Gal@glc\left(k\right)\times Intensity@glc\left(k\right)\right]}$$

$$\%Sia/Branch=\frac{\sum_{k=1}^{N}[\#Sia@glc\left(k\right)\times Intensity@glc\left(k\right)]}{\sum_{k=1}^{N}\left[\#GlcNAc@glc\left(k\right)\times Intensity@glc\left(k\right)\right]}$$

$$\%coreFuc=\frac{\sum_{k=1}^{N}[\#coreFuc@glc\left(k\right)\times Intensity@glc\left(k\right)]}{\sum_{k=1}^{N}Intensity@glc\left(k\right)}$$

where *N* represents the total number of glycoforms identified at this site, *#Gal@glc(k), #GlcNAc@glc(k), #Sia@glc(k), #coreFuc@glc(k)* represent the number of β-galactoses, branching GlcNAc (including bisected GlcNAc), sialic acids, and core fucoses in the glycan composition of the *k*^th^ glycoform, respectively. And *Intensity@glc(k)* represents the total reporter ion intensity from all associated tandem mass spectra of the *k*^th^ glycoform.

Because mannose and galactose cannot be distinguished without adequate glycan fragmentation coverage from oxonium ions, a single glycan composition could potentially be assigned to multiple glycan structures, thus often giving rise to an uncertain number of β-galactose moities. For instance, HexNAc(4)Hex(6) could be assigned to three glycan structures (M6A2, M5A2G1, and M4A2G2) containing zero to two β-galactoses, therefore the *#Gal|Glycan(k)* term in the equations, as well as the final glycan trait values, may be a range. In such a case, the average glycan trait value in that range was used for data analysis. Statistical analyses were performed using JMP software. For each N-glycosylation site, the difference of glycan traits between two genotype groups (N352S-WT) was evaluated through Student’s *t* test (n = 5).

## Results

### Quantitative Proteomic Analysis Reveals Limited Effects on Protein Expression or Secretion in B4GALT1 N352S Homozygotes

In order to improve the number of proteins identified in the undepleted human sera, basic-pH fractionation was conducted prior to the nano-LC-MS/MS analysis. A total of 500 protein genes were identified and 488 protein genes had detectable reporter ion signals in at least one sample. After excluding the genes corresponding to variable regions of immunoglobulins, 386 protein genes were included for final data interpretation (supplemental Table S2). In order to evaluate potential biases introduced during sample handling, including inaccurate protein amount estimation, unequal sample recovery during tryptic digestion and sample cleanup, and inequivalent sample pooling, we assessed the consistency of the sum of total reporter ion intensity from the major population of peptides across each TMT channel (see supplemental Fig. S2). To ensure unbiased evaluation, we excluded both the high-abundance peptides (intensity 1e7-1e11, representing approximately 20% of the total population but contributing to 98% of the total abundance) and the low-abundance peptides (intensity 1e3-1e6, which are subject to greater quantification uncertainty). The remaining medium-level peptides (intensity 1e6-1e7), which constitute a major portion of the peptide population, were used to evaluate the consistency of each sample. We observed that the sum of reporter ion intensity of these peptides was highly similar across all TMT channels (supplemental Fig. S2), indicating the absence of any significant bias prior to the sample mixing step. Therefore, no major normalization was required for downstream data interpretation.

**Fig. 2:**
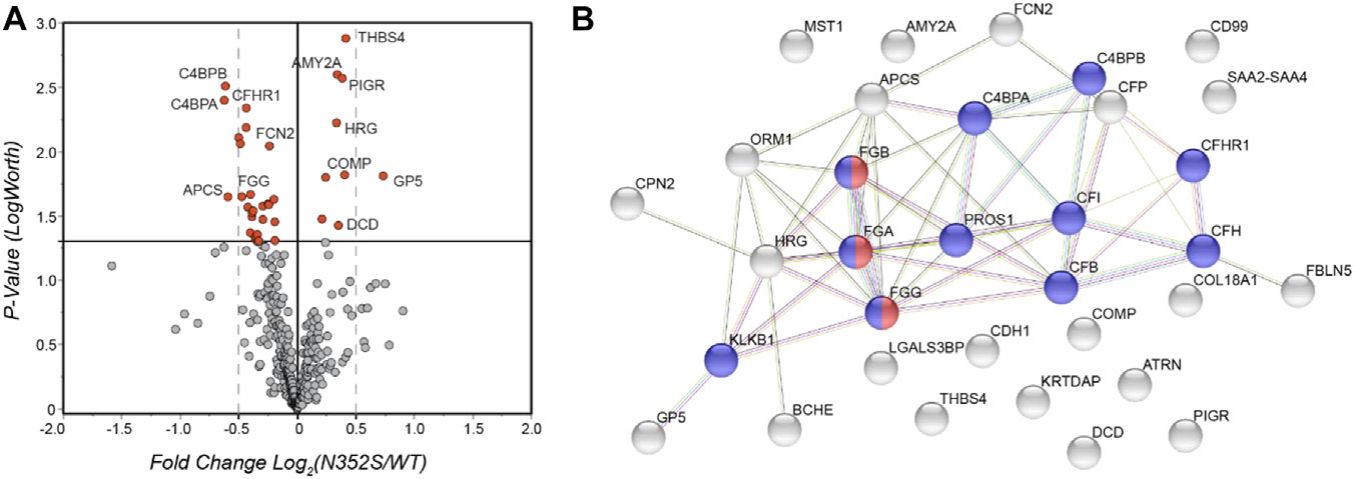
**Summary of quantitative analysis of proteome in human plasma samples.***A*, volcano plot of fold changes (logarithmic scale) in levels of plasma proteins in the *B4GALT1* WT and N352S genotype samples. A total of 33 polypeptide chains with significant fold changes (*p*-value < 0.05) are highlighted in *red*. *B*, a protein–protein interaction network of the 33 affected proteins highlighted in the volcano plot was generated through STRING analysis (string-db.org). Complement and coagulation-associated proteins are highlighted in *blue*, and fibrinogen is highlighted in *red*.

Among the 386 quantified plasma proteins, only 33 proteins were found to be significantly altered in minor homozygotes compared to WT, according to a *t* test with a *p*-value cutoff of 0.05 (Fig. 2*A*, and supplemental Table S2). This small portion (8.5%) with respect to the total proteins potentially decreased the quantification confidence, given the 5% false positive rate. No protein genes have shown significant differences between the two genotype groups using the FDR-adjusted *p* values (<0.05) (supplemental Fig. S3). This result indicated that N352S variant of *B4GALT1* might affect the protein levels of only a minority of protein groups, and the fold changes were small (0.7 < fold change < 1.3). Among the 33 affected proteins, all three chains of fibrinogen (FGA, FGB and FGG) were down-regulated in the N352S genotype samples, in agreement with our initial findings based on ELISA data (1). Fibrinogen is known to interact with 17 other affected proteins in this list (Fig. 2*B*). Beyond fibrinogen, the protein levels of a variety of other coagulation and complement pathway-associated proteins were modestly affected in the N352S genotype samples.

Although a lower LDL-c level in N352S genotype group was observed, gene enrichment analysis for these altered proteins did not identify any differences in levels of proteins in pathways involved in lipid metabolism, such as cholesterol synthesis and lipoprotein assembly. A previous study has also suggested a positive correlation between fibrinogens and the LDL/HDL ratio (23). In our shotgun proteomics data, the level of ApoB, which compose LDL particles was slightly lower in minor homozygotes, and the level of apolipoprotein A1 (ApoA1) which composes HDL particles was slightly higher, in agreement with the expected trend as well as the ELISA data (supplemental Table S3), these differences did not reach statistical significance (original *p*-values < 0.05). Several proteins that are directly involved in cholesterol synthesis, such as *cholesteryl ester transfer protein*, were also examined but did not show significant differences between genotypes. Many subtle differences might not have been identified in the proteomics data because of the small sample size (n = 5). In addition, the observed lower level of LDL-cholesterol might not have been correlated with the levels of apolipoproteins but more so with changes in protein function due to the altered N-glycosylation profiles.

### Glycosylation Profiles are Regulated at Many Glycosylation Sites in the B4GALT1 N352S Group

In the glycoproteomic dataset, a total of 151 glycoproteins were identified from the HILIC fractions containing enriched glycopeptides: 109 N-glycan compositions were found at 372 unique N-glycosylation sites, thus giving rise to 3183 unique glycoforms (supplemental Tables S4–S6). Example HCD MS/MS spectra of glycopeptides are shown in supplemental Fig. S4. Early HILIC fractions containing solely non-glycosylated peptides were also analyzed to evaluate potential biases in quantification between TMT channels during sample preparation and pooling, by following the same approach as described for the proteomics dataset. The evaluation pool comprising the medium-level peptides (excluding the first and last 20% of peptides ranked by abundance) from each TMT channel showed noticeable variation in the sum of total reporter ion intensity (supplemental Fig. S5). To address this, the sum of the total reporter ion intensity for each TMT channel was normalized to the same value, and the correction factors were thereby determined for each TMT channel. Then, the individual reporter ion intensities of glycopeptides identified from the glycopeptide-enriched fractions were adjusted using these correction factors (supplemental Fig. S5 and supplemental Table S6). In addition, a standard glycoprotein was spiked into the samples at various concentrations corresponding to 0.01% to 0.1% of the total plasma proteins for quality control purposes. This protein is a fusion of the second Ig domain of human vascular endothelial growth factor receptor 1 (FLT1) with the third Ig domain of human vascular endothelial growth factor receptor 2 (KDR) and contains two N-glycosylation sites from each domain. According to previous studies, the glycopeptides from endogenous FLT1 and KDR are not detected in the samples without spike-in; therefore, the endogenous proteins were not expected to substantially contribute to the glycoprofiles of the spiked-in standard glycoprotein. Glycopeptides from all four sites of the exogenous standard glycoprotein were successfully detected, and their identities were further verified through peptide mapping of pure standard glycoprotein samples when the matrix was not present, thus suggesting that the glycopeptide enrichment and nanoLC-MS/MS analysis workflow performed in the current study achieved sufficient data quality for site-specific N-glycosylation analysis of glycoproteins in human plasma samples.

**Fig. 3:**
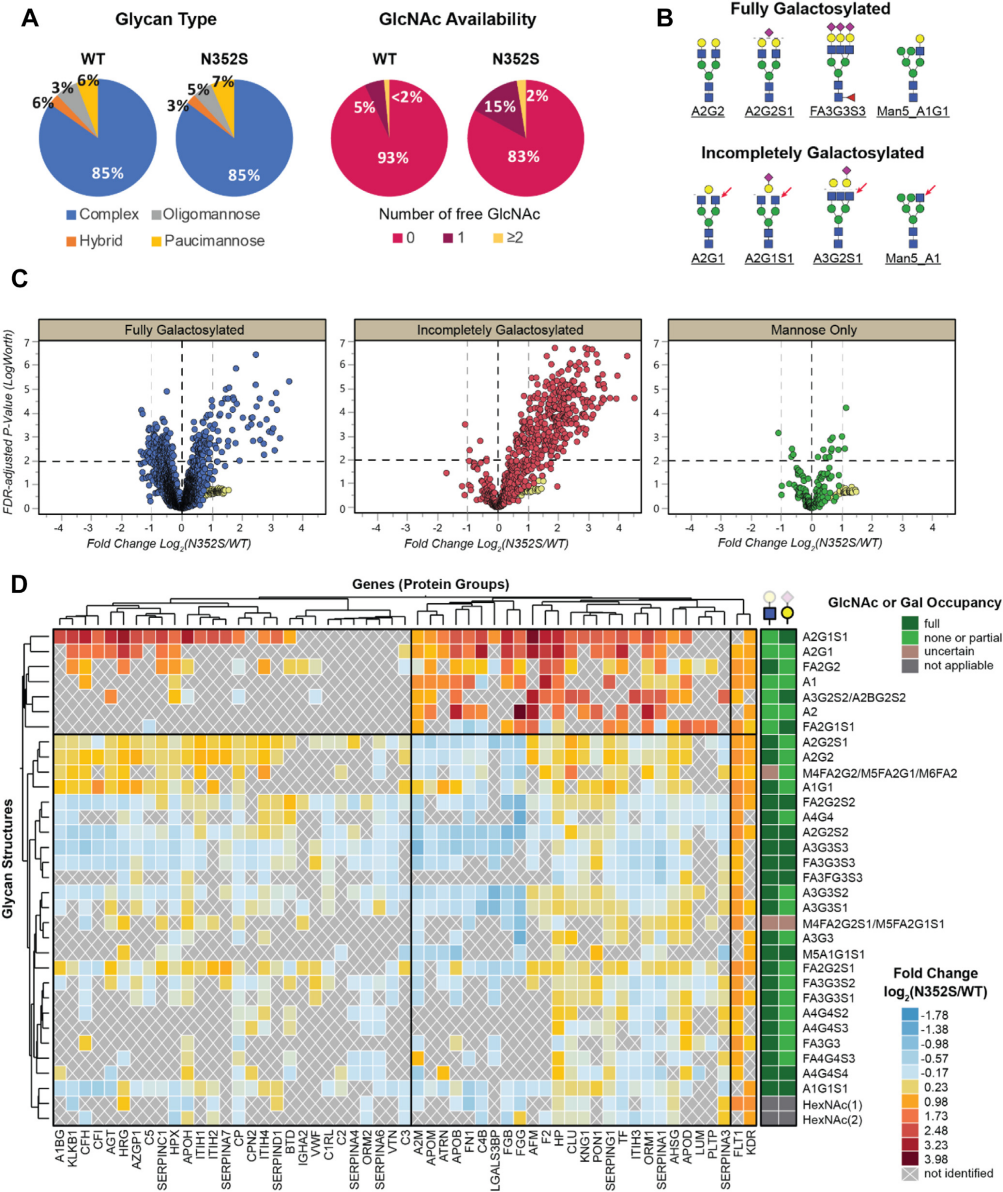
**Summary of quantitative analysis of intact N-glycoproteome in human plasma samples.***A*, glycan composition distribution within each B4GALT1 genotype group. The level of each glycan type was calculated on the basis of the total reporter ion intensity. *B*, example glycan structures for full galactosylation and incomplete galactosylation. *Red arrows* indicate the free terminal GlcNAc. *C*, volcano plots for all 3183 identified glycoforms, with false discovery rate (FDR)-adjusted *p*-value and the N352S:WT ratio of glycopeptide levels. Each point represents one glycoform. *Yellow dots* indicate the glycoforms from the spiked-in standard glycoprotein. All mis-cleaved peptides containing the same N-glycosylation site are combined. *D*, hierarchical clustering with heatmap and dendrograms of glycoforms distributed in different glycoproteins, of which the multiple glycosites were consolidated. Any glycoprotein gene (columns) that carried less than 10% of all glycans and any glycan (rows) that was not present on at least 10% of all glycoprotein genes were excluded. The status of galactosylation on GlcNAc or sialylation on galactose for each glycan is indicated in the last two columns.

Most secreted glycoproteins in human plasma are expected to contain complex-type glycans and to be highly galactosylated, because the galactosylated glycans are usually considered the mature forms contained in secreted proteins. Indeed, the glycan type distribution was highly similar between the genotype groups: complex-type glycans were observed in both genotype groups (Fig. 3*A*). Among the complex and hybrid-type glycans, fully galactosylated glycans were the major forms in both genotype groups; however, the N352S group exhibited a higher level of incompletely galactosylated glycans, which contain at least one free terminal GlcNAc. This elevation was likely to have been caused by the decreased amount of galactose transferred to GlcNAc in the N352S group as a result of the partial inhibition of the enzymatic activity of B4GALT1. Example structures of glycans with different galactosylation status are depicted in Figure 3*B*.

The differences in the abundance of all identified individual glycoforms between the N352S and WT groups are plotted in Figure 3*C*. Remarkably, the incompletely galactosylated complex/hybrid glycoforms containing at least one free terminal GlcNAc (as shown in the middle panel) exhibited a predominant upregulation in the volcano plot, in agreement with the expected lower occurrence of galactosylation in the N352S genotype group. In contrast, the fully galactosylated complex/hybrid glycoforms containing no free terminal GlcNAc exhibited changes in both directions although the upregulations appeared to be more pronounced (upper panel). For mannose-only glycoforms including oligomannose and paucimannose (bottom panel), fewer glycoforms showed significant differences in abundance with respect to the levels of complex- and hybrid-type glycoforms. These results indicated that the major fold changes in N-glycopeptides occurred on the partially galactosylated glycans. Most fully galactosylated glycoforms were expected to be downregulated in the N352S genotype samples because of the increased level of incompletely galactosylated glycoforms; however, many remained significantly up-regulated. Glycoforms from the spiked-in standard glycoprotein were present in a unique region with large *p* values (>0.05), but the large differences in levels, as displayed with yellow dots in Figure 3*C*, were consistent with the expected differences in spike-in concentrations among samples.

The altered glycan profiles of selected glycoproteins are presented in the hierarchical clustering heatmap shown in Figure 3*D*. This heatmap includes 55 glycoproteins that have at least ten glycoforms. In terms of glycan compositions, the top seven rows of the heatmap formed the most distinguishable cluster, exhibiting a strong up-regulation for most of the glycoprotein columns in the *B4GALT1* N352S genotype samples. This cluster contained all six incompletely galactosylated glycans (A2G1S1, A2G1, A1, A3G2S2, A2, and FA2G1S1) in the heatmap. On the other hand, the other rows of the heatmap, most of which were fully galactosylated complex or hybrid structures, such as A2G2 or M5A1G1S1, displayed diverse differences in levels in the N352S genotype samples. While these glycans were significantly down-regulated in some glycoproteins such as fibrinogen (FGB and FGG) due to the decreased amount of galactose transferred, they were mildly upregulated in other glycoproteins, such as inter-α-trypsin inhibitor heavy chain H4 (ITIH4) and clusterin (CLU). In addition, FLT1 and KDR originating from the spiked-in standard glycoprotein sample were clustered in the last two columns of the heatmap, due to their unique pattern of having all glycoforms equally increased in the N352S samples, consistent with the spiked-in concentrations. In summary, the hierarchical clustering heatmap revealed that the glycan structures were mainly clustered based on the occupancy status of GlcNAc, distinguishing between incompletely galactosylated and fully galactosylated.

**Fig. 4:**
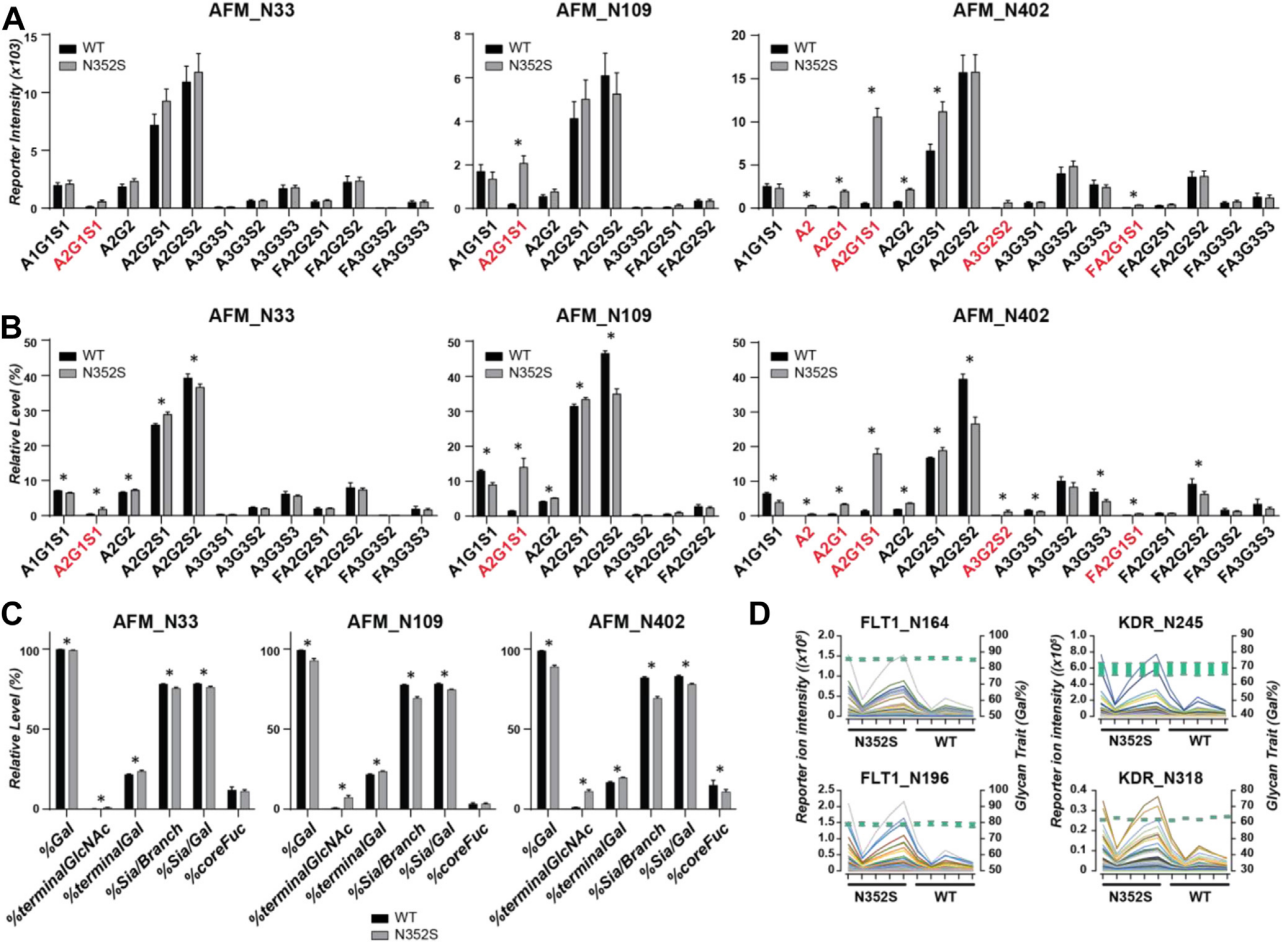
**Site-specific N-glycosylation profiles in a representative endogenous glycoprotein (afamin) and a spike-in standard glycoprotein.** N-glycosylation profiles in afamin for three identified N-glycosylation sites, shown as the calculated (*A*) absolute total reporter ion intensity for each glycoform, (*B*) relative levels with normalized total reporter ion intensity for each glycoform, (*C*) glycan traits for overall β-galactosylation, terminal saccharides, and core fucosylation. *Asterisks* indicate significant differences in *t* test. *D*, absolute total reporter ion intensity for individual glycoforms (*colored lines*) and calculated *%Gal* range (*green boxes*) at different sites of the spike-in standard glycoprotein across ten samples.

As presented in Figure 3, *C* and *D*, although the incompletely galactosylated glycoforms were widely up-regulated in the N352S genotype samples, the levels of corresponding fully galactosylated glycoforms, which were expected to be down-regulated, actually varied for different proteins. This variation might have been due to differences in protein expression or glycan occupancy or the presence of functional cross-talk in the glycan synthesis pathways between other glycosyltransferases (*e.g.*, for fucosylation, sialyation) and B4GALT1. More importantly, owing to the intrinsic abundance and diversity of fully galactosylated glycoforms in secreted proteins, as shown in Figure 3, the potential fold change for each of these glycoforms was smaller than those of the incompletely galactosylated glycoforms; these differences were also much smaller than the large individual variability across the five individuals in each group, as exemplified in three identified N-glycosylation sites of afamin (Fig. 4*A*). In contrast to the incompletely galactosylated glycoforms (highlighted in red), which had relatively low abundance and exhibited remarkable fold changes, the fully galactosylated glycoforms were high in both quantity and abundance; therefore, only subtle differences in levels were observed for most of these glycoforms, including the two most abundant ones (A2G2S1 and A2G2S2). The high variability among biological replicates further decreased the significance of the differences between genotype groups. Alternatively, the relative levels of individual glycoforms, calculated by normalization of individual glycoform abundance by the total abundance of all glycoforms detected at the same site of a given glycopeptide, eliminated the intra-group variability among the biological replicates; thus, a significant difference was observed between the genotype groups, with a low *p*-value (Fig. 4*B*). Interestingly, in all three glycosylation sites of afamin, among the fully galactosylated glycoforms, the doubly sialylated forms were always more abundant than the singly sialylated forms when other saccharide compositions in the glycoforms remained the same, such as A2G2S2 *versus* A2G2S1 or FA2G2S2 *versus* FA2G2S1. In addition, the difference was consistently higher in the WT group than the N352S group, thus suggesting that incorporation of sialic acid on galactose might also be diminished in the *B4GALT1* N352S genotype, thus giving rise to a relatively lower level of doubly sialylated glycoforms. Our findings also demonstrated that monitoring all individual glycoforms from the same glycosylation sites as a whole, and their abundance changes relative to one another, aid in elucidating site-specific glycan structure features associated with the *B4GALT1* variants.

The change in each glycan trait was strongly associated with the profiles of individual glycoforms. In afamin, for instance, the *%Gal* and *%terminalGlcNAc* from all three sites (Fig. 4*C*) changed as expected, owing to the change in relative distribution between the incompletely galactosylated and fully galactosylated glycoforms. The altered ratio between monosialylated and disialylated glycans was reflected by *%Sia/Gal*. This parameter can be used to evaluate whether sialylation activity is also affected because the absolute amount of galactose as the substrate of sialyltransferase is considerably diminished in the N352S genotype. In this case, although the activity of β-galactosylation (*%Gal*) was decreased in N352S samples, the occurrence of galactose at the terminal increased because of the decreased level of sialylation per galactose. Beyond the incorporation of sialic acid, a decreased *%coreFuc* was also observed at N402 in the N352S group. Whether the synthesis of core fucose can engage in cross-talk with B4GALT1 requires further investigation. Similar to the observations for individual glycoforms, the change in glycan traits for afamin N109 or N402 was much greater than that of N33, thus demonstrating that glycan traits can be used to quantitatively evaluate site-specific N-glycosylation profile changes. Glycan traits can also effectively eliminate the variation caused by absolute protein levels and/or glycosylation occupancy, as demonstrated in the analysis with standard glycoproteins spiked in the samples, in which a consistent trait value for %*Gal* was observed while the abundance of each individual glycopeptide varied considerably among samples (Fig. 4*D*).

**Fig. 5:**
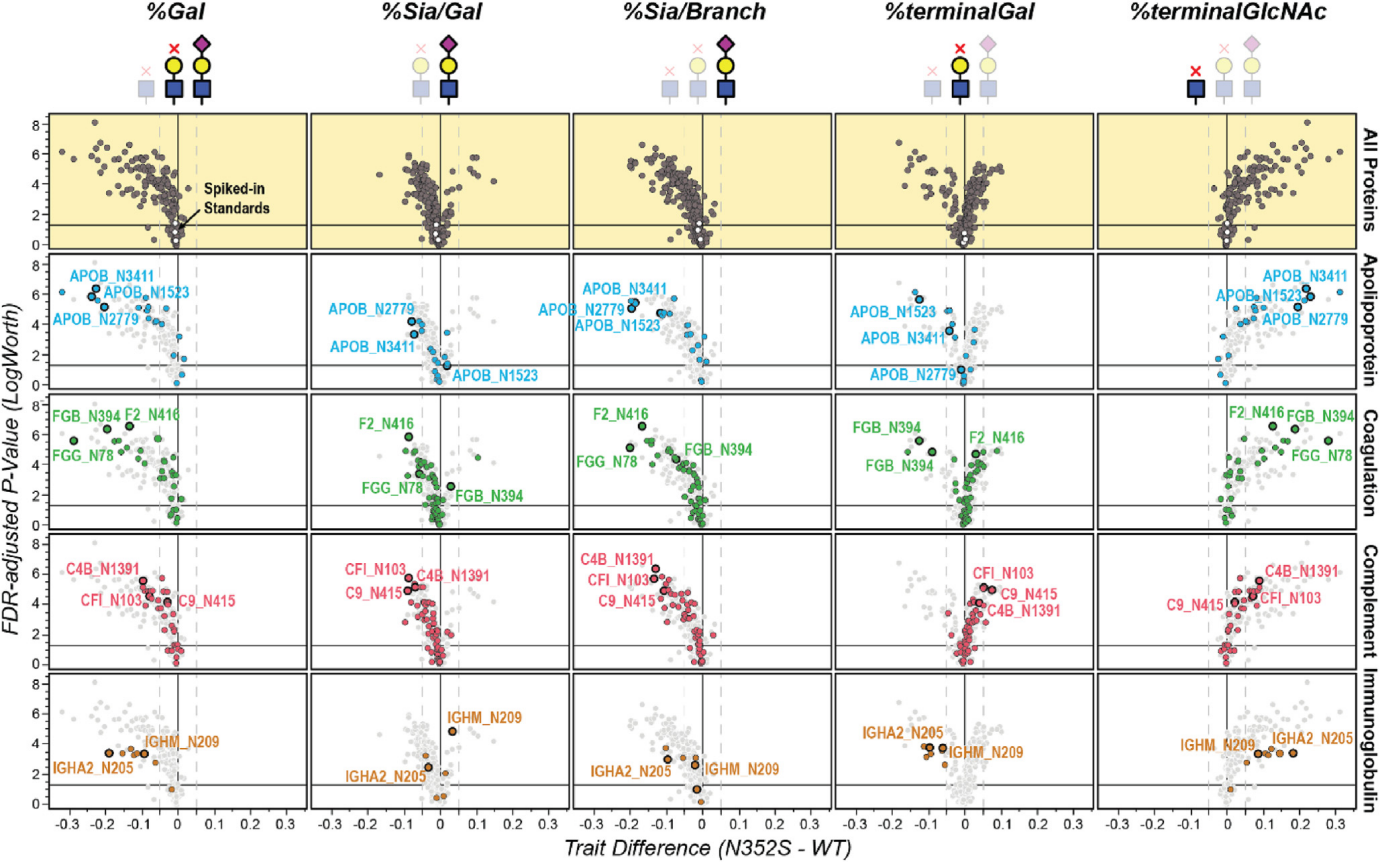
**Summary of site-specific glycan trait analysis.** Volcano plots of glycan traits, calculated based on site-specific N-glycosylation profiles for all identified glycoproteins (*top row*) as well as four protein categories of interest (rows 2–4, highlighted in a *different color*) in *B4GALT1* N352S and WT plasma samples were shown. Each *dot* represents the trait level change at one N-glycosylation site. Example N-glycosylation sites are labeled for each panel.

To quantitatively describe the changes in site-specific galactosylation and other glycan structure features, we calculated several glycan traits according to the combination of the relative abundance of individual glycoforms and their contribution to the structural features. The glycan trait of overall β-galactosylation (*%Gal*) from all identified N-glycosylation sites of proteins was screened based on their absolute differences and *p*-values adjusted according to the false discovery rate (Fig. 5). Out of the total 370 identified N-glycosylation sites, 168 sites exhibited 0% or 100% of *%Gal* as they were only occupied by glycans of high-mannose structure or fully galactosylated complex structure. Most of the remaining N-glycosylation sites showed a lower *%Gal* in the N352S genotype samples than the WT samples. In total, 67 sites from 42 proteins showed significant differences in levels (|difference| > 5%, *p*-value < 0.01). The N-glycosylation sites with a >15% difference are listed in Table 1. The *%Gal* for all identified N-glycosylation sites is shown in supplemental Table S7. The trend was not biased by the protein level, because no clear correlation was observed between the protein levels and the difference in *Gal%* (supplemental Fig. S6). Our results indicated that ApoB had the most glycosylation sites (N1523, N2779, N3411, N3465, N3895) at which significantly lower *%Gal* (differences <−20%) was observed in the N352S than the WT genotype. Fibrinogen (quantified as two separate chains, FGB and FGG) was another protein with significantly lower *%Gal* (differences <−19%) in N352S genotype samples. Full glycosylation profiles with relative levels of each individual glycoform identified on ApoB and fibrinogen are summarized in supplemental Figs. S7 and S8.

**Table 1:** Proteins containing N-glycosylation sites with >15% lower *%Gal* in the N352S than the WT samples

Gene name	Protein description	Site	*%Gal*		Protein level	
			Ave. diff. (N352S-WT)	*p*-value	Fold change (N352S/WT)	*p*-value
APOB	Apolipoprotein B-100	N1523	−23.6%	6.3e-08	0.8	0.32
		N2779	−20.1%	1.0e-06		
		N3411	−22.4%	5.0e-09		
		N3465	−31.8%	4.0e-08		
		N3895	−21.9%	2.0e-07		
ATRN	Attractin	N1082	−22.7%	1.6e-11	0.8	0.04
F11	Coagulation factor XI	N450	−17.2%	1.6e-07	0.9	0.06
FGB	Fibrinogen beta chain	N394	−19.2%	6.3e-09	0.8	0.03
FGG	Fibrinogen gamma chain	N78	−28.4%	2.5e-07	0.8	0.02
FN1	Fibronectin	N430	−21.2%	7.9e-10	0.7	0.16
IGHA2	Immunoglobulin heavy constant alpha 2	N205	−18.7%	6.3e-05	1	0.79
KLKB1	Plasma kallikrein	N396	−15.4%	2.0e-06	0.8	0.03
		N453	−16.1%	1.0e-07		
PCYOX1	Prenylcysteine oxidase 1	N353	−21.1%	2.5e-06	0.9	0.08
PZP	Pregnancy zone protein	N246	−16.2%	1.3e-05	0.7	0.32

*%Gal* directly or indirectly affected several terminal saccharide levels such as terminal GlcNAc, terminal GalNAc, terminal β-Gal, terminal α-Gal, and sialic acid. Because GalNAc and α-galactosylation were rarely identified in these human plasma samples, we only focused on the other three terminal saccharides interpreted in four terminal saccharide-associated glycan traits (*%terminalGlcNAc*, *%terminalGal*, *%Sia/Gal*, *%Sia/Branch*) through all identified N-glycosylation sites. As shown in the volcano plot (Fig. 5), *%terminalGlcNAc* was mainly up-regulated in the N352S genotype samples for most N-glycosylation sites, as opposed to *%Gal*. The *%Sia/Branch* was significantly down-regulated in the N352S genotype samples for most N-glycosylation sites, due to the decreased level of β-galactose to which sialic acid is attached. The overall decrease in sialic acid levels in the proteins might have significantly affected the protein net charge and protein function.

Although the N352S genotype samples exhibited exclusive downregulation of *%Gal* and the overall level of sialic acid (*%Sia/Branch*) across a broad range of proteins, both increased and decreased the percentage of galactose capped by sialic acid (*%Sia/Gal)* were observed in these samples, with the decreased *%Sia/Gal* being particularly remarkable (Fig. 5). This decrease in *%Sia/Gal* resulted in a potentially higher level of *%terminalGal* in the N352S genotype samples, as indicated by proteins involved in the coagulant pathway and the complement pathway. Conversely, a few key plasma proteins, including apolipoproteins (especially ApoB), immunoglobulins, and fibrinogens, exhibited decreased *%terminalGal* in the N352S genotype samples. Interestingly, our data, along with the previous study (1), revealed slight alterations in protein expression or secretion levels for many complement and coagulation-associated proteins including fibrinogen (Fig. 2; supplemental Tables S2 and S3), as well as immunoglobulins, ApoB in the N352S genotype samples. Therefore, the unique changes in *%terminalGal* observed in these proteins might provide valuable insights into the relationship between protein glycosylation and the signaling (*e.g.*, through lectin recognition) within relevant pathways, such as coagulation, immune response, and lipid metabolism. Furthermore, the N-glycosylation sites from the spiked-in standard protein exhibited no differences between the two genotype groups for any of these glycan traits, consistent with our expectation.

Terminal GlcNAc residues can be located either on glycan antennae or as bisecting structures. It is important to determine the impact of *B4GALT1* N352S on these two types of terminal GlcNAc, as they may exhibit opposite trends. In theory, only the antennary terminal GlcNAc can be the substrate of B4GALT1, and thus may be affected by the reduced enzymatic activity. To better understand the contribution of bisecting and antennary terminal GlcNAc to N-glycosylation profiles, it is necessary to distinguish between these two structural isomers. Although recent advances in software development and fragmentation optimization for sufficient oxonium ions have improved glycopeptide identification and guaranteed more accurate structure assignment (24), discrimination between these two isomers remains challenging. Unfortunately, the commercial software used in this study does not support this discrimination, and manual structure assignment based on insufficient oxonium ions is not reliable. To address this issue, we released N-glycans from each plasma and quantified the most abundant 69 plasma glycans (including linkage isomers) above our detection threshold (1e4). The methods were detailed in Supplemental Information. The structures with bisecting GlcNAc were confidently identified by using retention time under the HILIC condition and signature fragmentation ions (supplemental Table S8). We detected 15 bisecting GlcNAc-containing glycans, eight of which also contained a terminal GlcNAc residue located on branches. The total level of these glycans was elevated from 3.2% to 7.6% in *B4GALT1* N352S samples, as expected. In contrast, the total level of the remaining seven glycans, which only contain bisecting GlcNAc, was highly comparable in the two genotypes (3.7% in WT and 3.1% in N352S). We also examined four glycan compositions where both bisecting and tri-antennary isomers were detected. As shown in supplemental Fig. S9, all four tri-antennary glycans (A3G2, FA3G2S1, A3G3S1, A3G2S2), which contained one free terminal GlcNAc residue, were significantly increased in N352S samples, while their bisecting isomers (A2BG2, FA2BG2S1, A2BG2S1, A2BG2S2) were either significantly decreased or only modestly increased in *B4GALT1* N352S samples compared to the tri-antennary glycans.

The released glycan dataset was also used to calculate glycan traits, where bisecting GlcNAc was not considered to be available for further extension by galactose (see supplemental Fig. S9), which showed slight differences compared to the calculation without distinguishing between bisecting and antennary terminal GlcNAc. Nevertheless, the observed trends were still comparable, and they were consistent with the site-specific traits calculated based on glycopeptides, which included a lower *%Gal*, *%Sia/branch*, and an increased *%terminalGlcNAc* in the N352S genotype samples. The overall *%terminalGal* and *%Sia/Gal* were comparable between the two genotypes, only showing differences when site-specific trait analyses were achieved (Fig. 5). These findings suggest that the contribution of bisecting glycans to total incompletely galactosylated glycoforms was small in terms of the abundance: in the WT samples, the abundance of bisecting GlcNAc was only 24.6% of the total terminal GlcNAc, and this number was further reduced to 18.8% in the *B4GALT1* N352S samples because it was less affected by reduced B4GALT1 activity compared to terminal GlcNAc located at branches. In conclusion, these observations demonstrate that terminal GlcNAc was elevated in *B4GALT1* N352S samples only when it could be extended by galactose while bisecting GlcNAc may be resistant to level increase or even tend to be reduced in *B4GALT1* N352S samples due to the lack of available GlcNAc that can be extended by galactose.

## Discussion

Our data suggested that ApoB may be a key factor linking the altered glycosylation profiles to the lower levels of LDL-cholesterol observed in individuals with the N352S genotype. ApoB participates in the packaging of LDL particles, and its function is critical to the release of free LDL-c. Interestingly, the protein levels of ApoB showed no significant differences between the two genotypes (Table 1). Therefore, we hypothesized that the function of ApoB in LDL assembly (25) and/or ApoB-related LDL-c circulation in the blood or clearance by the liver may be directly influenced by the altered glycosylation profiles. This hypothesis could be further verified by profiling ApoB N-glycosylation in different-sized LDL particles and studying protein-protein interactions between ApoB and LDL receptors *in situ* or with recombinant proteins. For fibrinogen, the lower level of the *%Gal* and *%SiaPerBranch* in the N352S genotype samples might have caused the observed lower protein levels through attenuated secretion or faster clearance.

In this study, our proteomic study revealed that the protein levels of fibrinogen and several complement factors were slightly altered in the N352S genotype samples, compared with the WT samples. The N-glycosylation profiles at many sites across a variety of proteins were identified and quantified with TMT-based glycoproteomics. The glycan traits calculated based on the relative abundance of individual glycoforms at each site effectively overcame the challenges in the statistical analysis due to variations in protein levels and/or glycosylation occupancy among biological replicates within each genotype group, thus intuitively and quantitatively describing how the site-specific glycosylation profile is affected by the *B4GALT1* N352S missense variant, and providing insights into the relationship between glycan structures and protein function. The results revealed that levels of galactosylation and sialylation were diminished at almost all identified glycosylation sites of proteins in the N352S genotype samples. Moreover, proteins including ApoB and fibrinogen were the most affected targets, in agreement with the observed lower LDL-c and fibrinogen in individuals with *B4GALT1* N352S variants. In addition, the altered glycan traits, such as decreased sialyation per glycan branch, decreased terminal GlcNAc, and varying terminal galactosylation, may potentially affect the functions of proteins in many pathways (*e.g.*, complement activation) beyond lipid metabolism and coagulation. These effects should be monitored carefully if *B4GALT1* is pursued as a putative therapeutic target.

The major focus of this study was to investigate the effect of B4GALT1 on the galactosylation status of GlcNAc. A prerequisite of this strategy is precise structure assignment for each N-glycoform, which might be challenged by possible ambiguities in N-glycan structure assignment for the precursors with the same glycan composition. The first ambiguity arose from differentiating between bisecting GlcNAc and tri-antennary GlcNAc. Although site-specific identification through glycopeptides was not achieved in this study, globally released glycan data provided some insights that these two isomers may be affected in opposite ways by altered B4GALT1 activity. This is because galactose cannot be added to bisecting GlcNAc through β-1,4 linkage. The second ambiguity was distinguishing between common saccharides and those rarely reported in human plasma, such as GlcNAc and GalNAc or β-galactose and α-galactose. Our historical and released glycan data indicated undetectable levels of GalNAc and α-Gal-containing glycans in the clinical samples in this study, and therefore, they were not considered in data interpretation. While this may not be a concern for this study, it should be noted that the glycoproteomics workflow and data interpretation strategy outlined here may require significant revision when applied to other sample types, such as tissues, cell lines, or non-human samples, where the levels of GalNAc, or α-galactose may be higher.

Although the major conclusions were based on the protein glycosylation sites profiled in this work, these conclusions would be strengthened by analysis of additional weakly abundant proteins and/or more glycosylation sites. Although the observed effects on lipid metabolism were associated with the altered apolipoprotein glycosylation profiles, particularly that of ApoB, many other LDL metabolism-associated enzymes, such as cholesteryl ester transfer protein and proprotein convertase subtilisin/kexin type 9 (PCSK9), were not included in our proteomics and glycoproteomics analysis because of their low abundance. On the other hand, the calculation of galactosylation-related traits (*e.g.*, *%Gal*) relies on the detection of both fully galactosylated glycoforms and incompletely galactosylated glycoforms. As demonstrated in this study, the incompletely galactosylated glycoforms for most of the N-glycosylation sites were less abundant than other glycoforms; therefore, incompletely galactosylated glycoforms may not be captured, resulting in an overestimated level of galactosylation. In such cases, the results might not have reflected the true differences in glycosylation profiles between genotypes for those N-glycosylation sites.

Enhancing the detection sensitivity through more advanced sample enrichment or data acquisition methods would be key to increasing the likelihood of capturing and identifying low-abundance proteins and glycoforms in the plasma samples. For example, deeper depletion of highly abundant (*e.g.*, top 14 or 20) proteins could be used to further reduce the dynamic range of plasma samples and increase the relative abundance of low-abundance proteins (26, 27, 28). It has also been shown that extensively increasing the number of fractions during basic-pH fractionation can effectively improve protein identification in global protein analysis, even without protein depletion (29). Similarly, for glycopeptide analysis, collecting HILIC eluates in multiple fractions instead of a single-step HILIC enrichment can reduce the complexity and increase the number of identified glycopeptides (30). However, HILIC-based fractionation might be less efficient compared to reversed phase-based fractionation due to insufficient separation and too broad peak width through the HILIC column. In this study, indeed, exhaustive HILIC-based fractionation (every 0.5 min) did not significantly increase the number of identified glycopeptides compared to the runs with fewer number of fractionation (every 1 min). Instead, a secondary fractionation orthogonal to HILIC enrichment, such as basic-pH reversed-phase chromatography, may further reduce the sample complexity (31, 32). In addition to these general approaches, another strategy to include a positive control, such as the sample treated with enzymes that induce hydrolysis of β-1,4-galactose, along with other samples. Moreover, signal clean-up with high-field asymmetric waveform ion mobility spectrometry has also been demonstrated to improve the signal-to-noise ratio in detecting TMT-labeled glycopeptides (33).

## Data Availability

The raw files have been deposited in MassIVE (ID: MSV000090976).

## Supplemental Data

This article contains supplemental data.

## Conflicts of interest

The authors declare the following competing financial interests: M. E. M., A. R. S., and G. D. G. are inventors on US Patent Number 10,738,284 “B4GALT1 Variants And Uses Thereof” and international patent applications filed in Europe, Canada, New Zealand, Singapore, Israel, India, and Japan. A. R. S. and G. D. G. are inventors on a separate patent application related to this work filed by Regeneron Pharmadslceuticals, Inc, US Application No. 17/190,650, on 3 March 2021, which is pending. M. E. M. receives sponsored research support from Regeneron Pharmaceuticals, Inc. A. R. S. is an employee of Regeneron Pharmaceuticals, Inc and receives salary, stock and stock options as compensation. A. R. S. is also a part-time faculty member at the University of Maryland School of Medicine, in addition to his employment at Regeneron.
