# Supplementary material for: In-Depth Mass Spectrometry Analysis Reveals the Plasma Proteomic and N-Glycoproteomic Impact of an Amish-Enriched Cardioprotective Variant in B4GALT1
Source: Mol Cell Proteomics. 2023 Jun 15;22(8):100595. doi: 10.1016/j.mcpro.2023.100595 (PMC10392133; doi:10.1016/j.mcpro.2023.100595)
Supplement: Supplemental Information [file mmc2.docx]

**In-depth Mass Spectrometry Analysis Reveals the Plasma Proteomic and N-glycoproteomic Impact of an Amish Enriched Cardioprotective Variant in *B4GALT1***

Yunlong Zhao^1,*^, Shruti Nayak^1^, Shivkumar Raidas^1^, Lili Guo^1^, Giusy Della Gatta^2^, Sujeethraj Koppolu^3^, Gabor Halasz^3^, May E. Montasser^4^, Alan R. Shuldiner^2,4^, Yuan Mao^1,*^, Ning Li^1^

^1^Analytical Chemistry, Regeneron Pharmaceuticals, Inc., Tarrytown, NY 10591, USA.

^2^Regeneron Genetics Center, LLC, Tarrytown, NY 10591, USA.

^3^Molecular Profiling and Data Science, Regeneron Pharmaceuticals, Inc., Tarrytown, NY 10591, USA.

^4^Division of Endocrinology, Diabetes and Nutrition and Program for Personalized and Genomic Medicine, Department of Medicine, University of Maryland School of Medicine, Baltimore, MD 21201, USA.

**Supplemental Information**

**Supplemental Methods**

**Global analysis of released glycan**

The plasma samples were denatured and reduced in a solution containing 1% RapiGest^TM^ SF surfactant (Waters) and 5 mM TCEP, followed by digestion with PNGase F and labeling with RapiFluor-MS^TM^ fluorescence reagent (Waters). HILIC-based solid phase extraction cartridges (Waters) were used to purify the labeled glycans, which were then analyzed for N-glycan identification and relative quantitation using online liquid chromatography-mass spectrometry. The ACQUITY UPLC I-class system (Waters) coupled with a Q-Exactive Plus Hybrid Mass Spectrometer (Thermo Fisher Scientific) equipped with a HESI source was used to perform HILIC-MS in positive mode. Separation of glycans was achieved using an ACQUITY Glycan BEH Amide column (Waters, 2.1 mm × 150 mm) with a gradient shown in the table below:

| Mobile Phases | A: 50 mM ammonium formate (pH 4.4) | | | | |
| --- | --- | --- | --- | --- | --- |
|  | B: 85/15 (v/v) ACN/100 mM ammonium formate (pH 4.4) | | | | |
| Gradient | Time (min) | %A | %B | Flow (mL/min) | Gradient |
|  | 0 | 11.8 | 88.2 | 0.45 | Initial |
|  | 29 | 20.2 | 79.8 | 0.45 | Linear |
|  | 65 | 35.3 | 64.7 | 0.45 | Linear |
|  | 66.5 | 100 | 0 | 0.20 | Isocratic |
|  | 69.5 | 100 | 0 | 0.20 | Linear |
|  | 74 | 11.8 | 88.2 | 0.20 | Isocratic |
|  | 77 | 11.8 | 88.2 | 0.45 | Isocratic |
|  | 85 | 11.8 | 88.2 | 0.45 | Isocratic |

Full MS scans were collected from 400 to 2000 m/z, with a resolution of 70,000 and AGC target of 1e6. Data dependent MS2 scans were performed on the five most abundant precursors, with stepped NCE set to 16 and 28, and resolution set to 17,500. Other MS settings included a maximum IT of 250 ms, sheath gas of 40, aux gas of 10, sweep gas of 0, spray voltage of 3.8 kV, capillary temperature of 350 °C, aux gas heater temperature of 250 °C, and S-lens RF level of 50.

Glycans were manually identified by checking features with intensities above the specified threshold of 1e4. The precursor masses were matched with theoretical values, and the glycan structures were confirmed through fragmentation analysis using Xcalibur software (Thermo Fisher Scientific). Relative quantification was performed based on the extracted ion chromatographic (XIC) peaks of all identified precursors using Skyline Daily software (University of Washington). The total integrated XIC peak areas were exported to a spreadsheet for further data processing using Microsoft Excel.

**Supplemental Figures and Tables**

|  | **Wild type** | **Homozygotes** | **p** |
| --- | --- | --- | --- |
| **N** | 5 | 5 |  |
| **Sex** | 1M/4F | 1M/4F |  |
| **Age (years)** | 56.6 ± 14.1 | 51.4 ± 14.8 | 0.5 |
| **TC (mg/dl)** | 202.4 ± 13.5 | 193.6 ± 36.7 | 0.6 |
| **LDL (mg/dl)** | 123.2 ± 12.8 | 101.8 ± 34 | 0.2 |
| **HDL (mg/dl)** | 63.4 ± 12.5 | 73.2 ± 24.5 | 0.4 |
| **TG (mg/dl)** | 70.4 ± 58.8 | 91 ± 107.6 | 0.7 |

**Table S1. Basic and clinical characteristics of study samples. M: Male; F: Female; TC: Total cholesterol; LDL-c: Low density lipoprotein-cholesterol; HDL-c:High density lipoprotein-cholesterol; TG: Triglyceride.**

|  | **ELISA** | | **TMT-based LC-MS/MS** | |
| --- | --- | --- | --- | --- |
|  | **Ratio (N352 : WT)** | **-log10 pValue** | **Ratio (N352 : WT)** | **-log10 pValue** |
| **APOA1** | 1.2 | NA | 1.1 | 0.23 |
| **APOB** | 0.8 | NA | 0.8 | 0.48 |
| **Fibrinogen** | 0.8 | NA | 0.8 | 1.49 |
| **IgG** | 1.4 | NA | 1.1 | 0.56 |

**Table S3. Comparison between ELISA and TMT-based LC-MS/MS methods for the quantifying the proteins of interest (N352S/WT).**

**
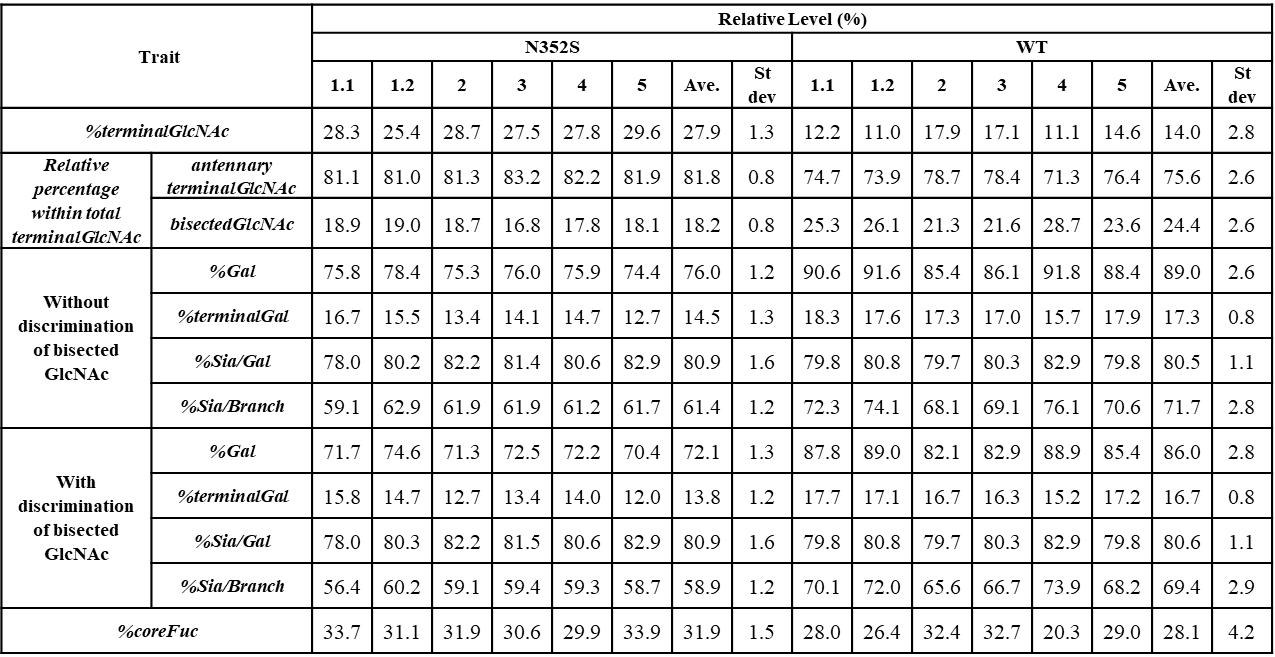
**

**Table S9. Glycan traits calculated from relative abundance of individual released glycans. 1.1 and 1.2 represent two technical replicates of a sample from each genotype.**

**
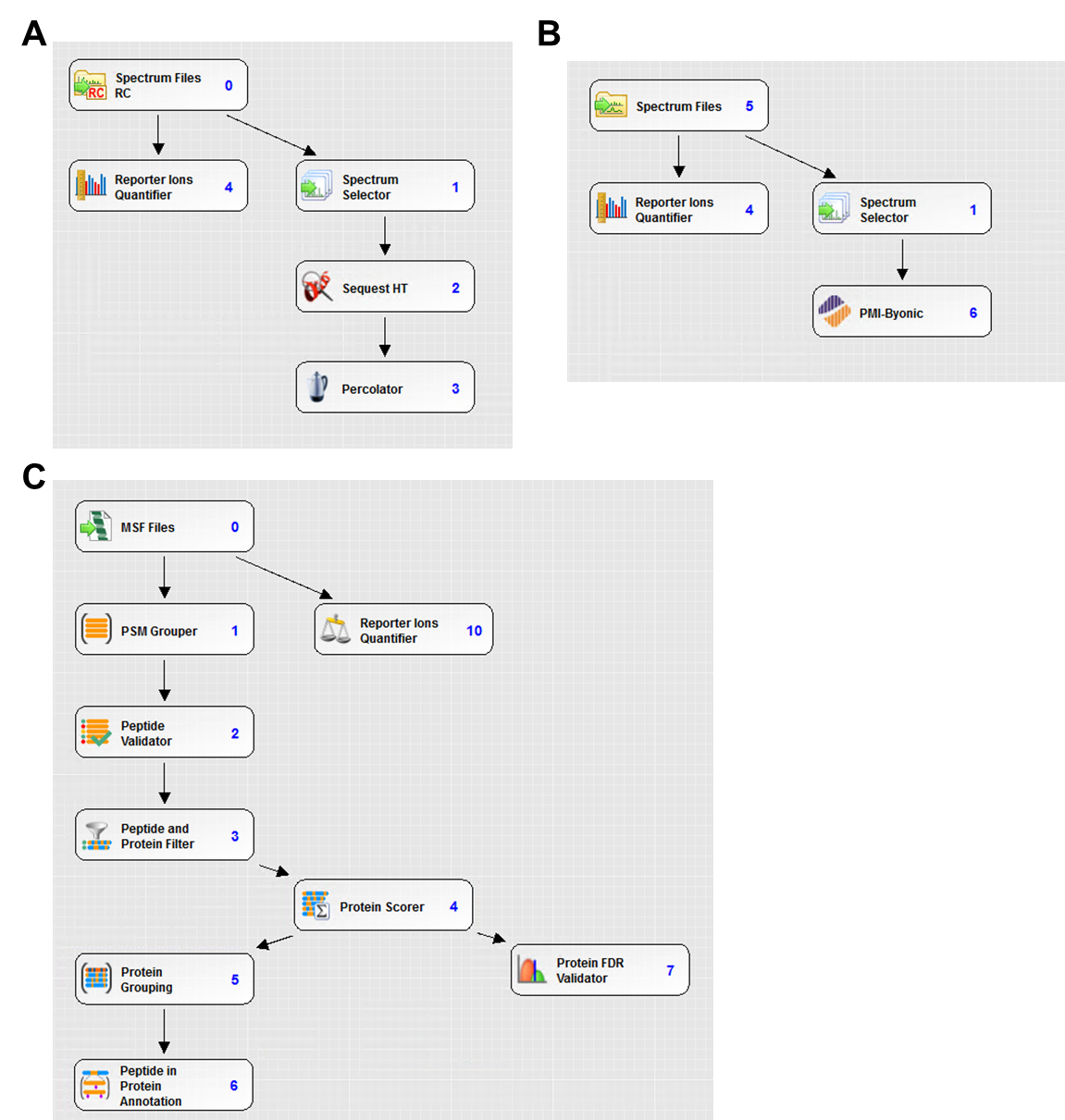
**

**Figure S1. Data processing workflow in Proteome Discoverer 2.2 (A) Processing step for proteomics dataset; (B) Processing step for glycoproteomics dataset; (C) Consensus step for proteomics and glycoproteomics datasets.**

**
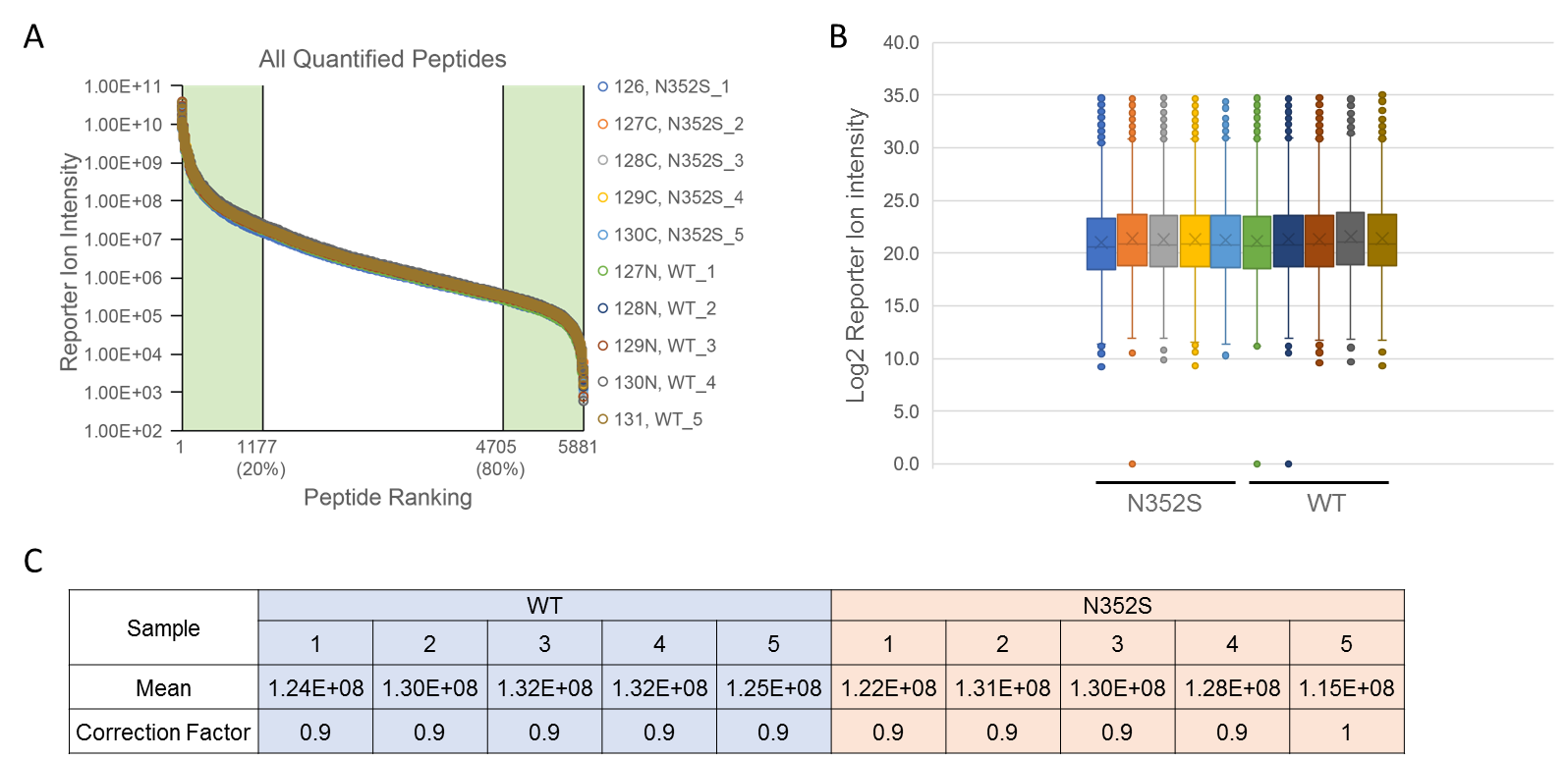
**

**Figure S2. Normalization of TMT reporter ion intensities in the proteomics dataset were performed by following steps. (A) All quantified peptides were ranked according to the TMT reporter ion intensity for each channel. Then, the first 20% and last 20% of peptides were excluded. And the remaining peptides were used as an evaluation pool for calculating correction factors. (B) Boxplot of reporter ion intensity from peptides in the evaluation pool from each channel for proteomics dataset was generated. Each box represents the interquartile range (IQR) defined by the 25^th^ percentile (Q1) and the 75^th^ percentile (Q3). The “-” and “×” symbols in the boxes indicate the median value and mean value for each sample, respectively. The error bars represent the minimum (Q1-1.5×IQR) and maximum (Q3+1.5×IQR). The data out of this range are shown as dots. (C) Corrected factors for each channel were determined by normalizing the mean values from each channel against the mean value from channel N352S_5.**


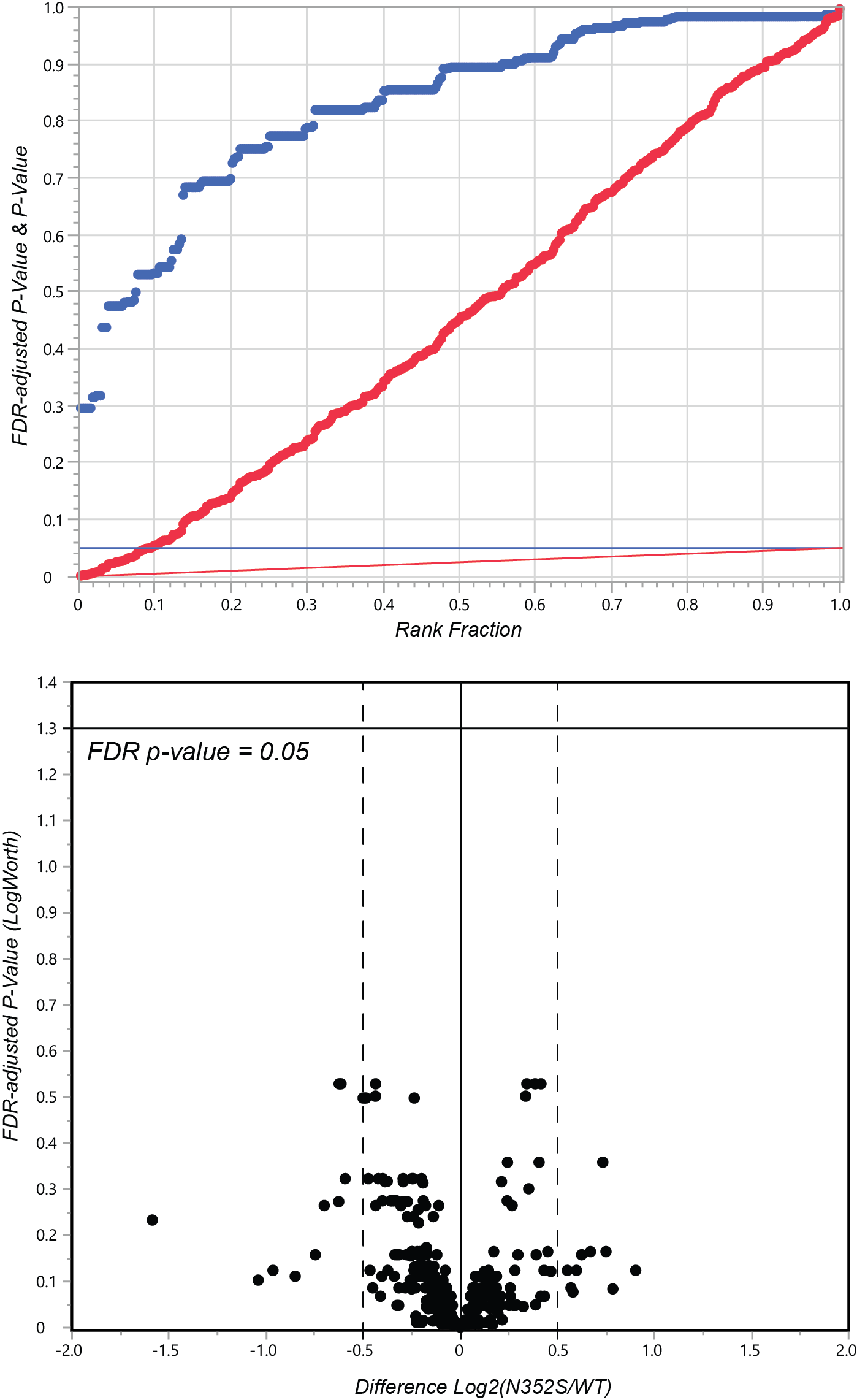


**Figure S3. Volcano plot for protein levels in the proteomics dataset using the P-Values with FDR adjustment.**


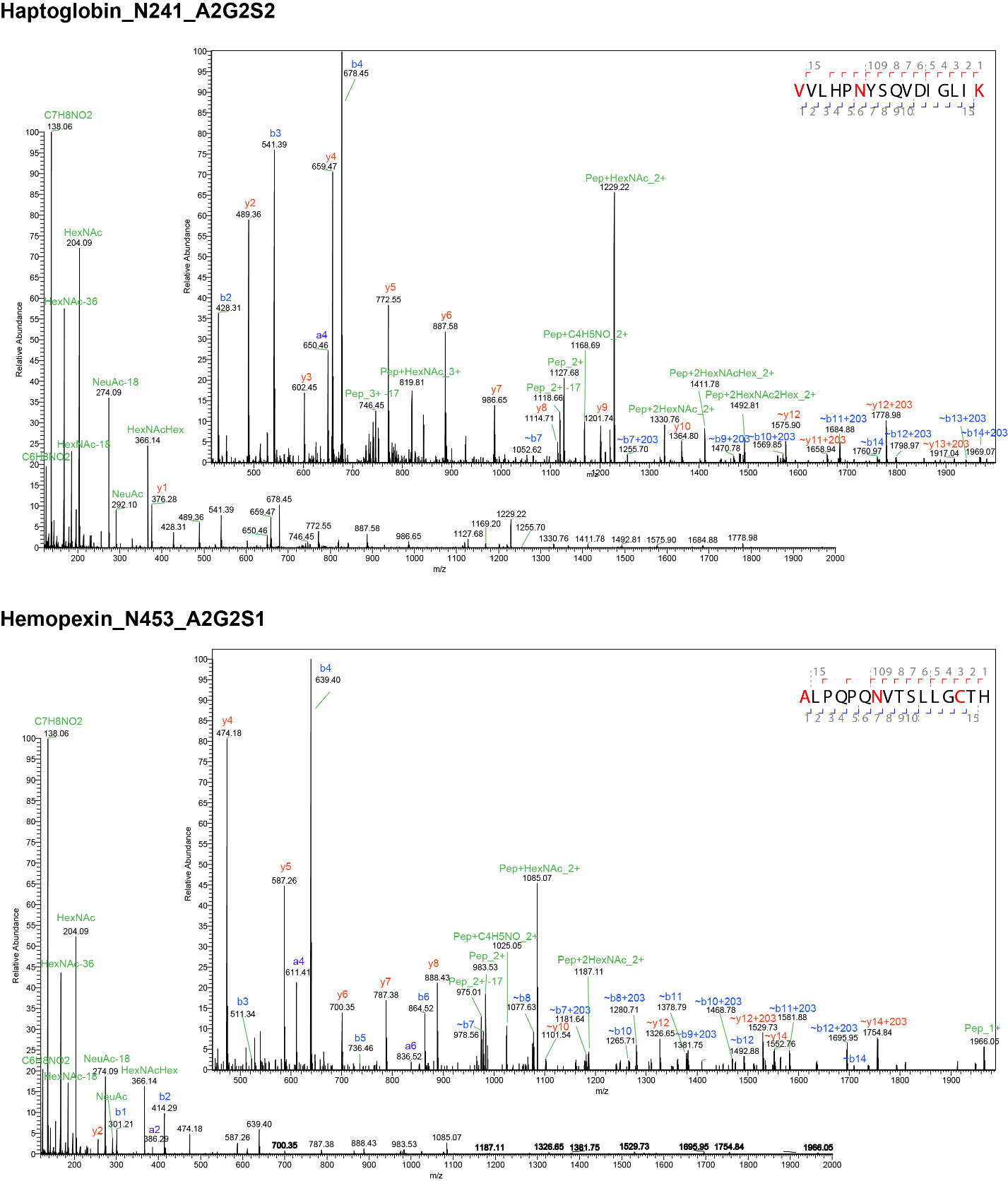


**Figure S4. Example HCD tandem spectra with fragmentation annotations. ~y and ~b represent the fragments with neutral loss of entire glycan.**

**
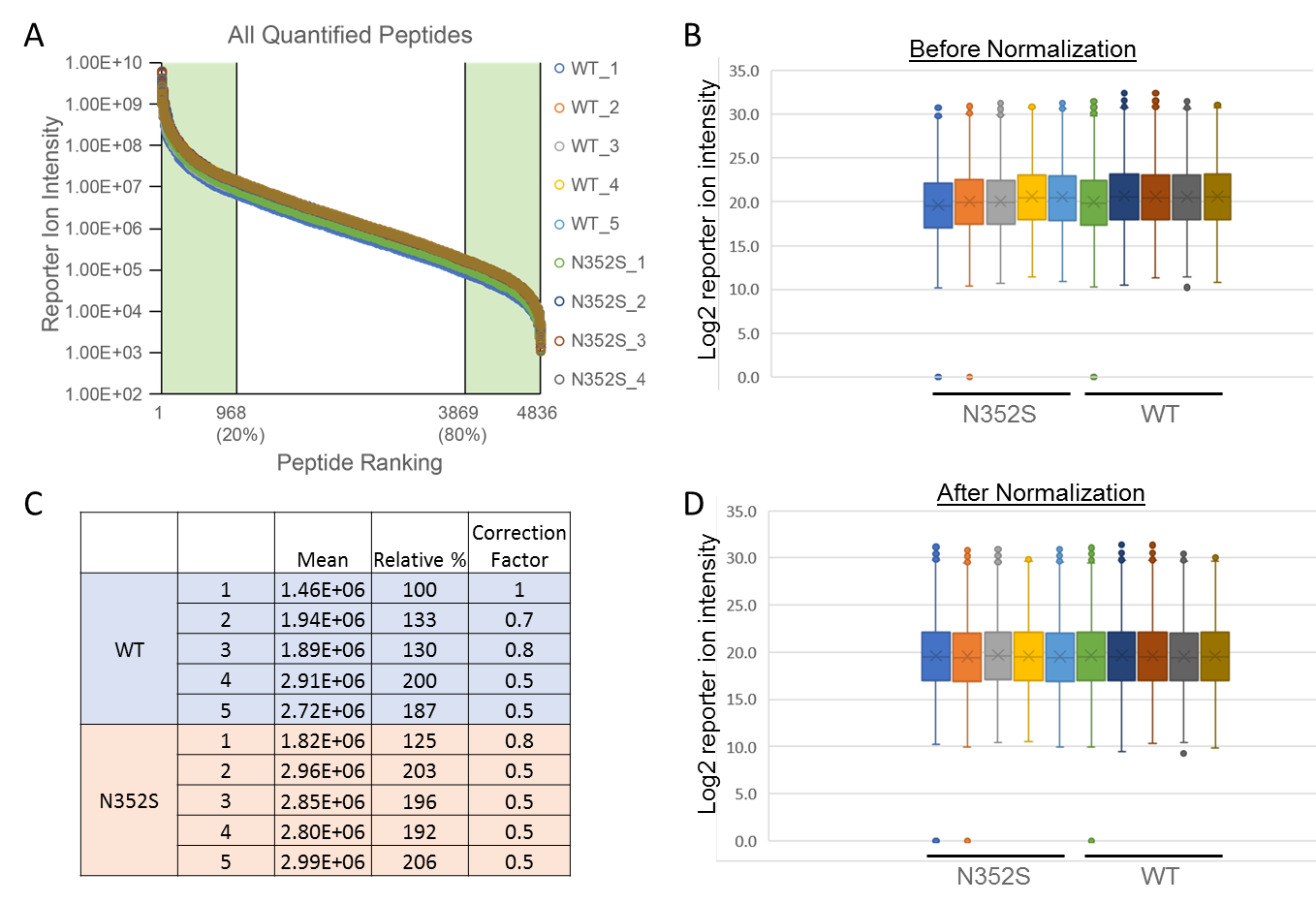
**

**Figure S5. Normalization of TMT reporter ion intensities in the glycoproteomics dataset were performed by following steps. (A) All quantified peptides from the early HILIC fractions solely containing non-glycosylated peptides were ranked according to the TMT reporter ion intensity for each channel. The peptides from spiked-in glycoproteins had been excluded. Then, the first 20% and last 20% of peptides were excluded. And the remaining peptides were used as an evaluation pool for calculating correction factors. (B) Boxplot of TMT reporter ion intensity from peptides in the evaluation pool from each channel was generated. Each box represents the interquartile range (IQR) defined by the 25^th^ percentile (Q1) and the 75^th^ percentile (Q3). The “-” and “×” symbols in the boxes indicate the median value and mean value for each sample, respectively. The error bars represent the minimum (Q1-1.5×IQR) and maximum (Q3+1.5×IQR). The data out of this range are shown as dots. (C) Corrected factors for each channel were determined by normalizing the mean values from each channel against the mean value from channel WT_1. (D) Boxplot was generated for the normalized TMT reporter ion intensity after applying the correction factors.**

**
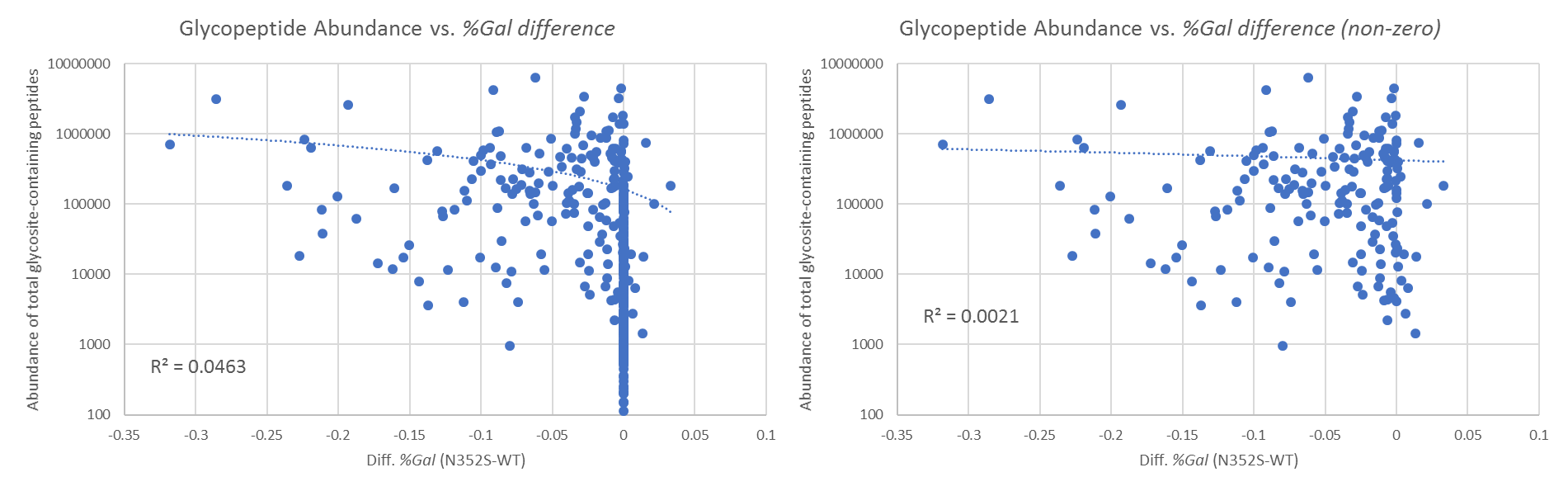
**

**Figure S6. Scatter plot of site-specific glycopeptide abundance vs. differnce of *%Gal* (N352S-WT) with or without data points of zero value. Each dot represents a single N-glycosite. The abundance was defined by the sum of total reporter ion intensity for all identified glycopeptides covering the desired N-glycosite, which may be related to protein abundance and glycan occupancy. The trends with linear regression fitting are indicated by the dotted lines. The poor R^2^ values suggest that there is no correlation between glycopeptide abundance and *%Gal* difference.**


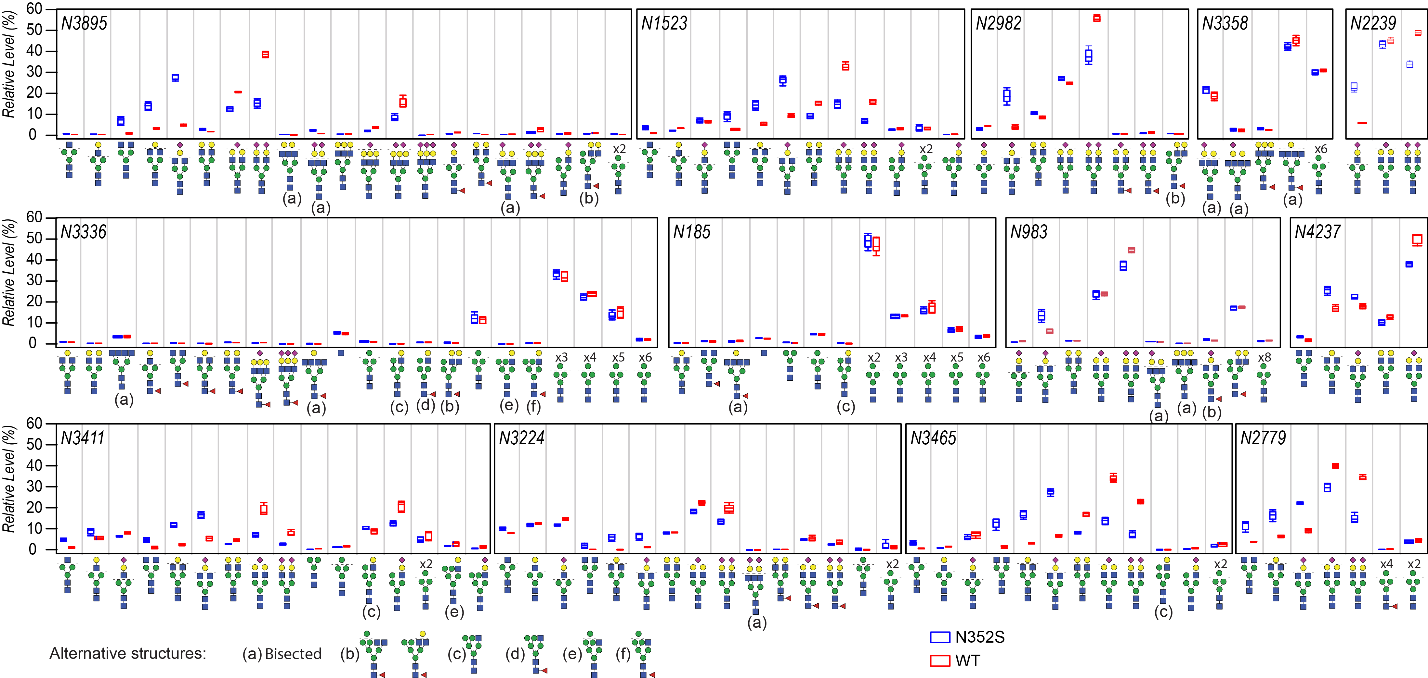


**Figure S7. Glycoforms for all identified N-glycosylation sites at apolipoprotein B-100 (ApoB). Each box represents the normalized level of individual glycoform relative the total glycoforms in WT (red) or N352S (blue), quantified using the sum of reporter ion intensity from all PSMs of individual glycoforms (including any miss-cleaved version of peptide and any metal ion adduct version of glycan) with |LogProb| > 2 and MS/MS Score > 200.**

**
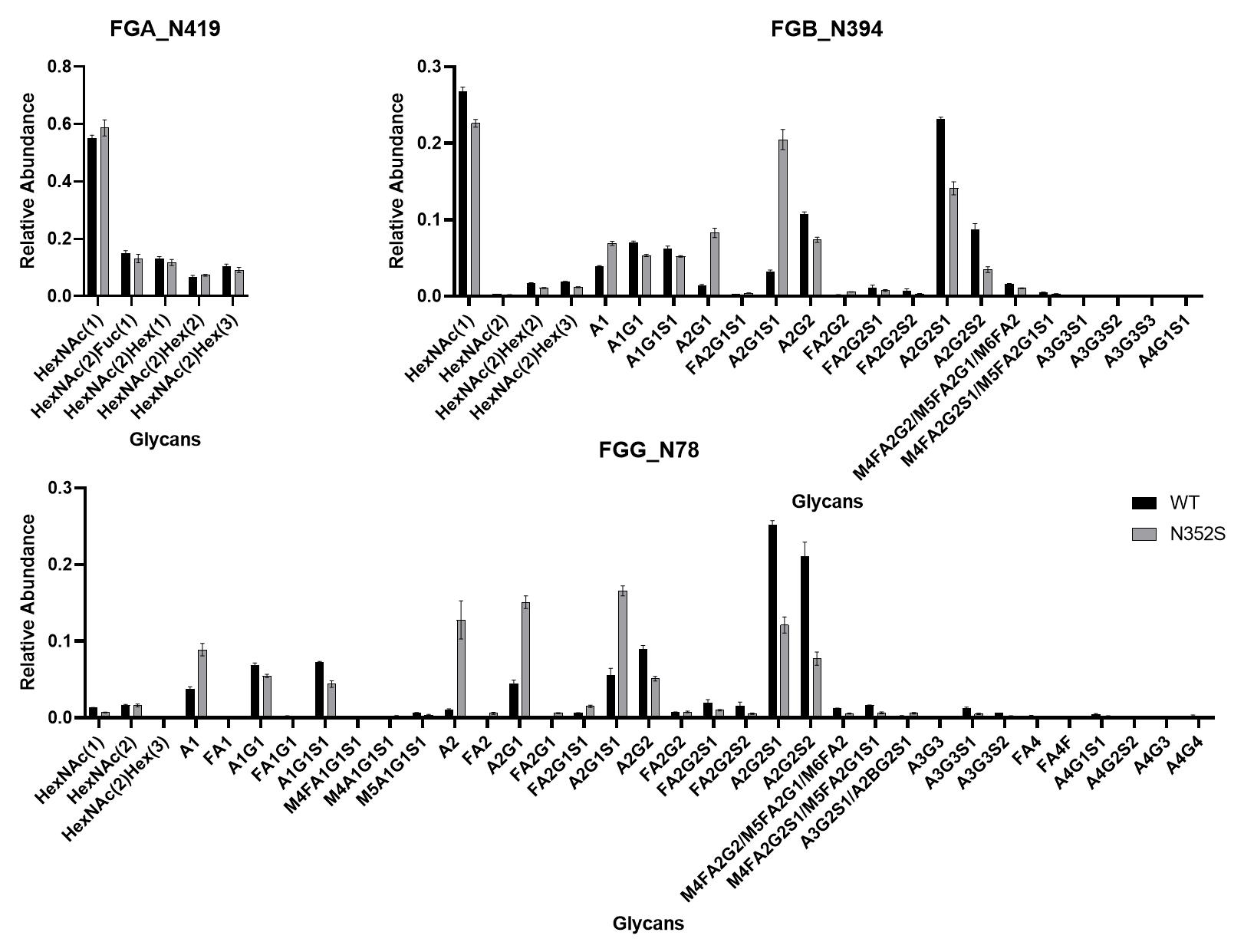
**

**Figure S8. Glycoforms for all identified N-glycosylation sites at three fibrinogen chains (FGA, FGB, FGG). Each bar represents the normalized level of individual glycoform relative the total glycoforms in WT or N352S, quantified using the sum of reporter ion intensity from all PSMs of individual glycoforms (including any miss-cleaved version of peptide and any metal ion adduct version of glycan) with |LogProb| > 2 and MS/MS Score > 200.**

**
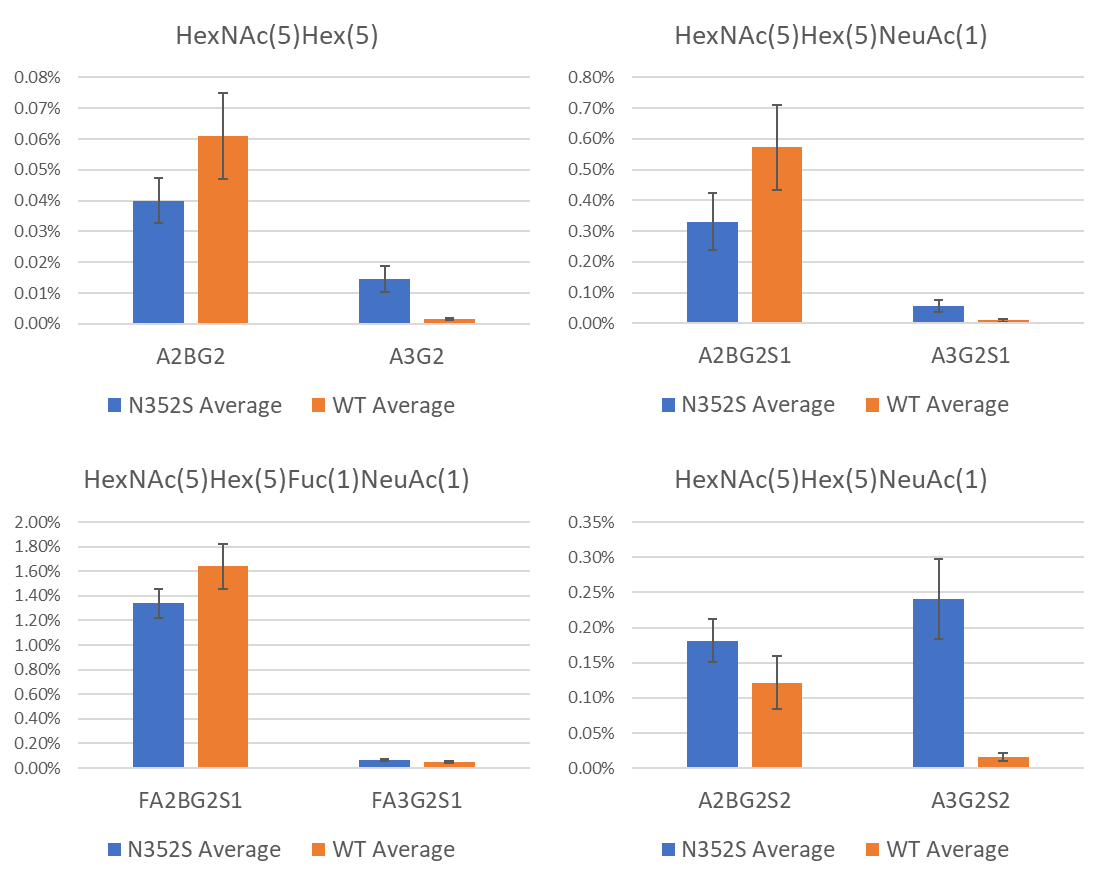
Figure S9. Comparison between level of bisecting and tri-antennary isomers containing one terminal GlcNAc. The level was quantified based on released glycans.**
